# A genome‐edited *N. benthamiana* line for industrial‐scale production of recombinant glycoproteins with targeted N‐glycosylation

**DOI:** 10.1002/biot.202300323

**Published:** 2023-10-15

**Authors:** Benjamin Kogelmann, Stanislav Melnik, Michaela Bogner, Somanath Kallolimath, Eva Stöger, Lin Sun, Richard Strasser, Marc‐André D'Aoust, Pierre‐Olivier Lavoie, Pooja Saxena, Johannes S. Gach, Herta Steinkellner

**Affiliations:** ^1^ Department of Applied Genetics and Cell Biology University of Natural Resources and Life Sciences Vienna Austria; ^2^ acib – Austrian Centre of Industrial Biotechnology Vienna Austria; ^3^ Medicago Inc. Quebec Quebec Canada; ^4^ Division of Infectious Diseases, University of California, Irvine Irvine California USA

**Keywords:** CRISPR/Cas9, fucose, N‐glycan engineering, Nicotiana benthamiana, recombinant glycoproteins

## Abstract

Control over glycosylation is an important quality parameter in recombinant protein production. Here, we demonstrate the generation of a marker‐free genome edited *Nicotiana benthamiana* N‐glycosylation mutant (NbXF‐KO) carrying inactivated β1,2‐xylosyltransferase and α1,3‐fucosyltransferase genes. The knockout of seven genes and their stable inheritance was confirmed by DNA sequencing. Mass spectrometric analyses showed the synthesis of N‐glycans devoid of plant‐specific β1,2‐xylose and core α 1,3‐fucose on endogenous proteins and a series of recombinantly expressed glycoproteins with different complexities. Further transient glycan engineering towards more diverse human‐type N‐glycans resulted in the production of recombinant proteins decorated with β1,4‐galactosylated and α2,6‐sialylated structures, respectively. Notably, a monoclonal antibody expressed in the NbXF‐KO displayed glycosylation‐dependent activities. Collectively, the engineered plants grow normally and are well suited for upscaling, thereby meeting industrial and regulatory requirements for the production of high‐quality therapeutic proteins.

## Graphical Abstract and Lay Summary

Engineering glycosylation pathway in plants increases quality of recombinantly expressed proteins. A *Nicotiana benthamiana* line that lacks active xylosyltransferase genes was used to knock out core fucosyltransferase genes by multi‐target CRISPR/Cas9‐based gene editing. The engineered line synthesizes human‐type GnGn structures well suited for industrial‐scale production of proteins with homogeneous N‐glycans and serves as template for further glycan diversifications.

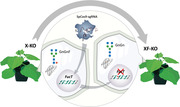

## 1 INTRODUCTION

Plants have been used for the expression of recombinant proteins for about three decades, prompting several interesting market products.^[^ ^1^ ^]^ In particular, the development of transient expression tools that allow for the rapid production of complex proteins within days of delivery of the DNA construct to plant leaves has significantly advanced the system.^[^ ^2^ ^]^ *Nicotiana benthamiana* plants are highly suited for transient expression and, as previously demonstrated, can produce monoclonal antibodies in multimeric formats.^[^ ^3^, ^4^, ^5^, ^6^ ^]^ These proteins are usually heavily glycosylated with significant impacts of this central post‐translational modification on biochemical and functional features.^[^ ^7^ ^]^ Therefore, controlling glycosylation is an important quality parameter in recombinant protein production. While plants synthesize complex N‐glycans similar to humans, they are simpler and less diverse. In addition, plant N‐glycans carry β1,2‐xylose and core α1,3‐fucose residues usually absent on mammalian proteins. Moreover, it was shown that IgG antibodies with eliminated fucose exhibit increased effector functions.^[^ ^7^ ^]^

Recent developments in genome editing (i.e., CRISPR/Cas9) have created new possibilities for genomic manipulations. While efficient protocols exist for plants with diploid genomes, targeted gene editing is more challenging in complex genomes, like *N. benthamiana* with an allotetraploid genome.^[^ ^8^ ^]^ Although CRISPR/Cas9 technology was applied to this plant species (e.g.,^[^ ^9^, ^10^ ^]^), established protocols for efficient editing are still limited. Notably, one study reports the knock‐out of active β1,2‐xylosyl‐ and core α1,3‐fucosyltranferase genes and N‐glycans of a recombinantly expressed IgG antibody were free of xylose and fucose.^[^ ^9^ ^]^ However, other proteins with more complex glycosylation were not characterized, or additional human typical glyco‐engineering, such as β1,4‐galactosylation or sialylation, was not performed. Thorough characterization of glyco‐engineered lines is necessary for their widespread adoption as universal expression platforms and will help to assess their suitability for the introduction of further modifications and to exclude unexpected effects on the N‐glycan structures observed in other model organisms.^[^ ^11^ ^]^

The present study is focused on the generation of a *N. benthamiana‐*based industrially suitable expression line synthesizing N‐glycans lacking β1,2‐xylose and core α1,3‐fucose. This refers to the inactivation of at least six genes that code for β1,2‐xylosyl‐ and core α1,3‐fucosyltranferases *(NbFucT1‐ NbFucT4*, *NbXylT1 and NbXylT2*). An additional gene *NbFucT5* is present, which may be a pseudogene.^[^ ^12^ ^]^ A TALEN‐edited *N. benthamiana* line (NB14‐29aT2) with inactivated *XylT* genes (*NbXylT1 and NbXylT2*) and two *FucT* genes (*NbFucT1 and NbFucT2*), but carrying three unmodified *FucT* genes served as starting plant line.^[^ ^13^ ^]^ Here we report the consecutive stacking of CRISPR/Cas9‐mediated mutations on top of those generated by TALEN, in order to knock out *NbFucT3, 4* and *5*. We primarily selected plants that carry homozygous frameshift mutations and are free of any transgene sequences. Mass spectrometry was used for a detailed elucidation of glycan composition of endogenous proteins and several reporter proteins, with intricate glycosylation features. Additionally, we focused on the controlled elongation of engineered N‐glycans towards typical human structures.

## 2 MATERIALS AND METHODS

Materials and Methods used in this study can be found in the Supporting information.

## 3 RESULTS AND DISCUSSION

### 3.1 CRISPR/Cas9‐mediated knockout of *NbFucT* genes

**FIGURE 1:**
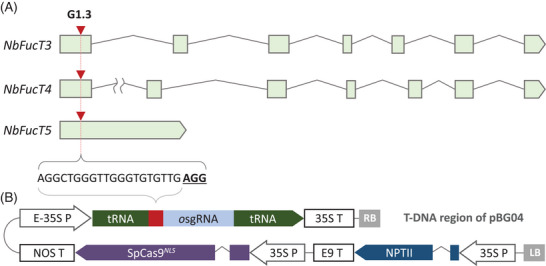
Illustration of CRISPR/Cas9 construct. (A) Schematic presentation of the three α‐1,3‐fucosyltransferase (*NbFucT3‐5*) genes targeted. Position of the double strand break induced by SpCas9/G1.3 complex is represented with a red triangle. Target nucleotide sequence (with protospacer adjacent motif underlined) is identical among the three genes and the matching spacer sequence of the guide G1.3. (B) Transformation vector pBG04 (T‐DNA region, not to scale): E‐35S P – enhanced CaMV 35S promoter; tRNA ‐ tRNA^Gly^ from *Arabidopsis thaliana*; *o*sgRNA – optimized sgRNA scaffold with extended hairpin.^[^ ^14^ ^]^; 35S T, E9 T, NOS T: respective CaMV 35S, rbcS and nopaline synthase gene terminator; NPTII – neomycin phosphotransferase II; SpCas9*^NLS^ Streptococcus pyogenes* Cas9 with nuclear localization signals; LB and RB – T‐DNA left and right border.

NB14‐29aT2, a TALEN edited *N. benthamiana* line, was used as starting line.^[^ ^13^ ^]^ Apart from four inactivated genes (*NbFucT1*, *NbFucT2*, *NbXylT1* and *NbXylT2*), three *FucT* genes (*NbFucT3, NbFucT4* and *NbFucT5*) remained unmodified and most probably active in this line. Several sgRNAs simultaneously targeting all three unedited *NbFucT* genes were designed to match perfectly the respective target sequences at the upstream exons while minimizing potential off‐target sites within the *N. benthamiana* genome. Transient evaluation of gene‐editing activity was performed (Tables S4 and S3) and the most efficient construct (pBG04) was used for subsequent stable cotyledon transformation (Figure 1). Regenerants were transferred to soil and the edited *NbFucT3* and *NbFucT4* genes were confirmed by molecular methods (see Figure S2). Four double gene edited lines were identified and further screened for edits in *NbFucT5*. Finally, after several screening rounds, one individual plant (NbBG04‐4) that revealed edits in the three assigned *FucT* genes and did not carry gene‐editing and antibiotic resistance transgenes, was selected (see Figures S4 and S5).

Amongst NbBG04‐4 T1 progeny (obtained by selfing) an individual plant NbBG04‐4‐18‐14 with homozygous frame‐shifting mutations in all five *FucT* genes, and lacking gene‐editing and antibiotic resistance transgenes, was selected for further characterization. This line is unique in that all introduced mutations, originating from both TALEN‐ and CRISPR/Cas9, occur close to the N‐terminus of respective proteins, minimizing the length of potentially translated polypeptides (Table S5). Stability of mutations was monitored until T3, the line assigned as NbXF‐KO.

### 3.2 Characterization of NbXF‐KO line

**FIGURE 2:**
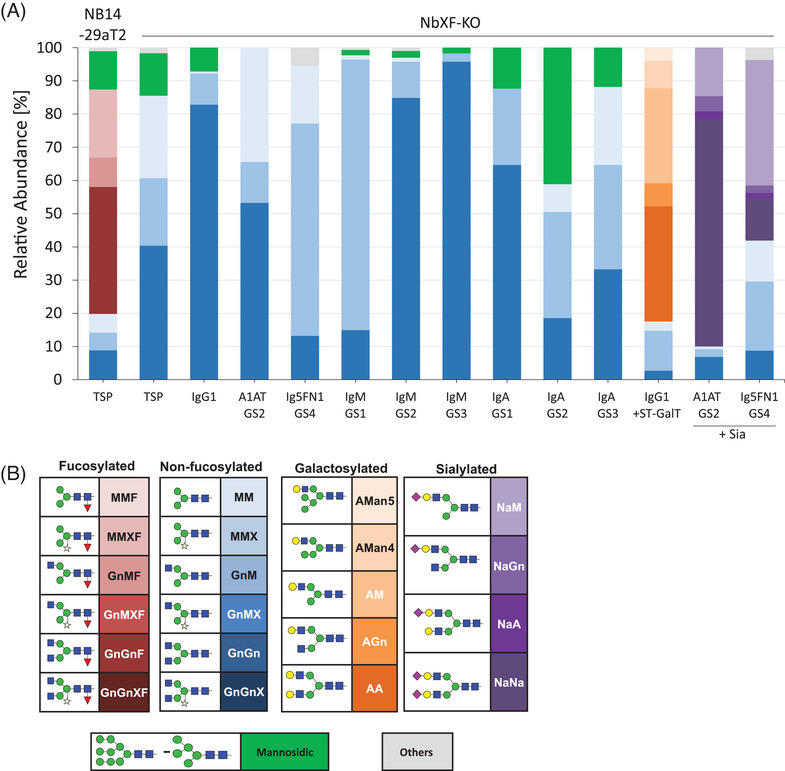
N‐glycan composition of endogenous proteins and recombinantly expressed glycoproteins. (A) Relative quantification of N‐glycans of total soluble proteins (TSP) and recombinantly expressed reporter proteins. IgG1: Immunoglobulin G1 (rituximab); A1AT: human α1‐antitrypsin, Ig5FN1: module of human neural cell adhesion molecule (NCAM); IgM: Immunoglobulin M; IgA: Immunoglobulin A2m; GS: glycosite; ST‐GalT: co‐expression of IgG1 with modified human β1,4‐galactosyltransferase^[^ ^24^ ^]^; +Sia: co‐expression of glyco‐reporter with multi‐gene vector PICH88266 carrying six genes for introducing the human sialylation pathway into plants.^[^ ^26^ ^]^ NB14‐29aT2 represents progenitor line. TSP represent average of 6 independent T4 NbXF‐KO plants, IgG1 represents 4 independent T4 plants. For further details see Figure S8 and Tables S8, S9 and S10. (B) Schematic representation of glycoforms according to Consortium for Functional Glycomics (www.functionalglycomics.org).

NbXF‐KO plants were cultivated under controlled growth conditions and did not exhibit any phenotypic alterations compared to WT plants. MALDI‐TOF analyses of N‐glycans released from total soluble proteins (TSP) exhibited ∼85% complex N‐Glycans (GnGn/GnM/MM) along with mannosidic structures (∼15%) (Figure 2, Table S9). No fucose‐carrying N‐glycans were detected, in contrast to the progenitor line NB14‐29aT2 that exhibited ∼67% fucosylated N‐glycans (Figure 2). Notably, MALDI‐TOF spectra indicated the presence of 1%–3% fucose containing Lewis A structures (α1,4‐linked fucose) (Figure S8). These results confirm the inactivation of all active core α1,3‐fucosyltransferase genes in the NbXF‐KO line.

### 3.3 Expression of recombinant glyco‐reporter in the NbXF‐KO line

Five human glycoproteins were transiently expressed in NbXF‐KO plants: monoclonal antibodies (IgG1, IgA2, and IgM), the enzyme inhibitor α1‐antitrypsin (A1AT) and a domain of the human neural cell adhesion molecule (Ig5FN1). These proteins carry xylose‐ and fucose‐containing N‐glycans when expressed in WT *N. benthamiana*.^[^ ^13^, ^15^, ^16^, ^17^ ^]^ The antibodies were immunopurified from total leaf extracts, whereas A1AT and Ig5FN1 were isolated as apoplastic fluid fractions. Glycosylation profiles of purified recombinant proteins (Figure S6), determined by liquid chromatography‐electrospray ionization‐tandem mass spectrometry (LC‐ESI‐MS), displayed a single dominant glycoform, namely, xylose‐ and core fucose‐free GlcNAc‐terminated structures (predominantly GnGn), accompanied by mannosidic structures that varied depending on the reporter and the glycosite (Figure 2, Table S9). Fucosylated N‐glycans were not detected. Compared to WT plants, no obvious constraints in expression of recombinant proteins were noticed, with yields of purified Abs between 150–300 mg/kg leaf material. Batch‐to‐batch variation of expression levels was between 10%–15%. No differences in glycan profiles or protein assembly were observed (Figure S9).

Previously an *N. benthamiana* RNAi‐based *XylT* and *FucT* knock‐down mutant (ΔXT/FT)^[^ ^18^ ^]^ which is widely used by academia and industry, for example,^[^ ^19^, ^20^, ^21^, ^22^, ^23^ ^]^ was developed. A direct comparison of the glycosylation profiles of glyco‐reporters produced in NbXF‐KO and ΔXT/FT revealed significant amounts of fucose (up to 25%) on some ΔXT/FT produced proteins, for example, IgA and IgM (Figure S7, Table S10). These results illustrate the high quality of the NbXF‐KO line.

### 3.4 Augmentation of the glycosylation repertoire using NbXF‐KO line

The NbXF‐KO line was further evaluated for its capacity to support further N‐glycan‐engineering. One important extension of the GnGn structures frequently observed on mammalian proteins but absent in plants is β1,4‐galactosylation. Co‐expression of the human β1,4‐galactosyltransferase (ST‐GalT)^[^ ^24^ ^]^ with IgG1 resulted in the synthesis of fucose‐free efficiently galactosylated N‐glycans (up to 80% AA, AGn, AM). An additional, hence more challenging glyco‐engineering approach, aimed at the synthesis of α2,6‐sialylated N‐glycans. This requires the *in planta* transfer of the human sialylation pathway, involving the coordinated overexpression of six foreign genes.^[^ ^25^ ^]^ For this we used a multigene vector (PICH 88266) that carries the six missing genes^[^ ^26^ ^]^ for its co‐expression with IgG1, A1AT and Ig5FN1, respectively. Importantly, IgG1‐Fc requires the presence of core fucose for efficient sialylation,^[^ ^27^ ^]^ thus a core α1,3‐fucosyltransferase was co‐expressed in this case. LC‐ESI‐MS glyco‐profiles of all the reporter exhibited up to 80% sialylated N‐glycans (Figure 2, Figure S7). In contrast, no fucosylated structures were detected (except for sialylated IgG1, where core fucose was present) and no constraints in the expression of recombinant proteins compared to WT plants were observed. The results confirm the applicability of the NbXF‐KO plants for complex glyco‐engineering.

### 3.5 Functional activity of monoclonal antibodies produced in XF‐KO

**FIGURE 3:**
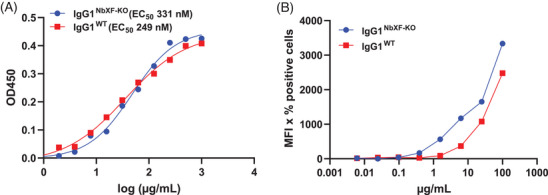
Functional activities of IgG1. (A) Antigen binding ELISA of purified rituximab (Rx) expressed in NbXF‐KO and WT.  (B) Median Fcγ‐receptor binding affinity against the TZM‐bl FcγRIIIa receptor (F158 allotype); rituximab expressed in NbXF‐KO and WT plants (IgG1^NbXF‐KO^ and IgG1^WT^).

Finally, functional activities of NbXF‐KO‐ and WT‐derived reporters were evaluated. IgG1 was monitored for its antigen and Fcγ‐receptor binding capacities. FcγRIIIa was chosen since its binding is significantly influenced by the presence/absence of core fucose.^[^ ^28^ ^]^ ELISA experiments show similar antigen binding of both Ab glyco‐variants (EC50 249 nM and 331 nM, respectively, Figure 3A). Contrary to WT, the NbXF‐KO‐produced IgG1 exhibited increased binding to FcγRIIIa (Figure 3B) in the cell‐based assay, as expected for IgG1 Abs with reduced core fucosylation. These results emphasize the high quality of the NbXF‐KO‐expressed proteins.

Collectively, we demonstrate the knock‐out of seven genes in *N. benthamiana* plants using multiplexed CRISPR/Cas9 approach building on a TALEN edited line, and the inactivation is stably inherited. By the combination of different reported techniques, we established an efficient CRISPR/Cas9‐based procedure adopting cotyledon transformation and prior experimental evaluation of sgRNA. This allowed the selection of a sgRNA with high editing efficiency, while several sgRNAs have been applied with varying transformation efficiencies.^[^ ^9^ ^]^ To avoid the possibility of subsequent off‐target mutations and to ensure genetic stability and consistency of the host plant line, we selected progeny carrying the desired homozygous or biallelic knockout mutations in all target genes but lacking the SpCas9 transgene. Transgene elimination also renders our host plant line suitable for further stacking of traits by the sequential combination of mutations, and it may facilitate the propagation and distribution of the host plant line in compliance with GMO regulations. The results are in line with previous studies that report the successful inactivation of *XylT* and *FucT* genes in the same plant or in *N. tabacum*,^[^ ^9^, ^29^ ^]^ however it is not clear whether these lines are free of any foreign sequences.

Notably, GnGn, the major N‐glycan species synthesized in NbXF‐KO is the conserved N‐glycan species in higher eukaryotes and serves as substrate for most N‐glycan diversifications in mammals. Here, we demonstrate the controlled elongation of GnGn towards galactosylated and sialylated N‐glycans. This can hardly be achieved in other expression platforms and is especially challenging in mammalian cells due to the presence of a complex endogenous glycosylation machinery which involves a large metabolic network of several hundred proteins that orchestrate the large glycan diversity.^[^ ^30^ ^]^ Finally, batch‐to‐batch consistency of recombinant proteins demonstrates the high profile of the NbXF‐KO line, thereby meeting the WHO standards for the production of high quality biotherapeutics.^[^ ^31^ ^]^

## 4 CONCLUSION

Genome editing was used to generate NbXF‐KO plants displaying complex N‐glycans completely devoid of plant‐specific β1,2‐xylose and core α1,3‐fucose residues. NbXF‐KO plants might contribute to an increased availability of high value biopharmaceutical products. This is especially interesting, as GnGn is the conserved complex N‐glycan species of higher eukaryotes and serves as template for greatest diversification. This also applies to the abundantly galactosylated and sialylated human proteins. In addition, factors such as relative cost effectiveness and simple handling might contribute to a more equal distribution of such products also in the low‐ and middle‐income countries.

## AUTHOR CONTRIBUTIONS

Benjamin Kogelmann: data curation, formal analysis, investigation, project administration, validation, visualization, writing – original draft, writing – review and editing; Stanislav Melnik: data curation, formal analysis, investigation, methodology, validation, visualization, writing – original draft, writing – review and editing; Michaela Bogner: formal analysis, investigation; Somanath Kallolimath: data curation, formal analysis, visualization, writing – review and editing; Eva Stöger: conceptualization, supervision, writing – review and editing; Lin Sun: investigation, formal analysis, writing – review and editing; Richard Strasser: conceptualization, funding acquisition, writing – review and editing; Marc‐André D'Aoust: conceptualization, data curation, funding acquisition, supervision, writing – review and editing; Pierre‐Olivier Lavoie: conceptualization, data curation, supervision; Pooja Saxena: data curation, supervision; Johannes S. Gach: data curation, formal analysis, investigation, writing – review and editing; Herta Steinkellner: conceptualization, funding acquisition, supervision, writing – original draft.

## CONFLICT OF INTEREST STATEMENT

During the study M.‐A.D., P.‐O.L. and P.S. were employed by Medicago. The remaining authors declare that the research was conducted in the absence of any commercial or financial relationships that could be construed as a potential conflict of interest.

## Supporting information

Supporting Information

## Data Availability

The data that supports the findings of this study are available in the supplementary material of this article.
