# Supplementary material for: A genome‐edited N. benthamiana line for industrial‐scale production of recombinant glycoproteins with targeted N‐glycosylation
Source: Biotechnol J. 2023 Oct 15;19(1):2300323. doi: 10.1002/biot.202300323 (PMC11475529; doi:10.1002/biot.202300323)
Supplement: Supplementary file 1 — Supporting Information [file BIOT-19-2300323-s001.pdf]

Supporting information for

A genome-edited *N. benthamiana* line for industrial-scale production of recombinant glycoproteins with targeted N-glycosylation

Benjamin Kogelmann[a, b], Stanislav Melnik[a, b], Michaela Bogner[a], Somanath Kallolimath[a], Eva Stöger[a], Lin Sun[a], Richard Strasser[a], Marc-André D'Aoust[c], Pierre-Olivier Lavoie[c], Pooja Saxena[c], Johannes S. Gach[d] and Herta Steinkellner[a,*]

[a] Department of Applied Genetics and Cell Biology, University of Natural Resources and Life Sciences, Vienna, Austria

[b] acib - Austrian Centre of Industrial Biotechnology, Muthgasse 18, 1190 Vienna, Austria.

[c] Medicago Inc., Quebec, QC, Canada

[d] University of California, Irvine, Division of Infectious Diseases, Irvine, CA,

*Correspondence:

Herta Steinkellner

Department of Applied Genetics and Cell Biology, University of Natural Resources and Life Sciences, Vienna, Muthgasse 18, 1190 Vienna, Austria

Tel: +43-1-47654-94370,

Fax: 43-1-47654-94009,

Email: herta.steinkellner@boku.ac.at

Material and Methods

Design of gRNAs

Sequences of at least 20 nucleotides long in homologous exonic or exon-intron interface regions of *NbFucT3* and *NbFucT4* identified as identical between the two genes (EMBOSS Needle, https://www.ebi.ac.uk/Tools/psa/emboss_needle/) and followed by SpCas9 PAM were considered as potential targets only when the respective sgRNAs did not form strong secondary structures in the spacer region (RNAfold, http://rna.tbi.univie.ac.at/cgi-bin/RNAWebSuite/RNAfold.cgi) and there were no off-targets within other known genes of *N. benthamiana* genome (CCTop, https://cctop.cos.uni-heidelberg.de:8043/). Accession of the targeted genes are listed in Table S2.

Construct design and cloning

Three multiplex constructs (pDV14, pDV15, pBG03, see Figure S1) containing SpCas9 under CaMV 35S promoter and up to 5 tRNA-sgRNA-tRNA units arranged in one cluster and driven by CmYLCV promoter were assembled as described recently [1], except pYLCV2 and pOGS2 (both featuring sequences allowing for tRNA- instead of Csy4-mediated processing of resultant polycistronic transcript) served as templates for amplification of sgRNA modules, and another destination plasmid pB357D with a different SpCas9 expression unit lacking the CDS for Csy4 was used in the final assembly step. Guides were distributed between different constructs such that each construct received only guides whose target sites on genomic DNA stayed >60 nt apart and there were no more than 2 guides per amplicon for unambiguous interpretation of DNA sequencing data when analyzed by TIDE. For the construction of pBG04 used for stable transformation of *N. benthamiana* and generation of knockout lines, pTTR1 in place of pYCLV2 was used as template for amplification of the upstream module of the sgRNA cassette. The binary vector pB357D is a derivative of B357p9ioR-35sCasWT (DNA Cloning Service, Germany) with tRNA sequence found in pOGS2, pYLCV2 and pTTR1 originating from the same plasmid. The CmYLCV and enhanced CaMV 35S promoters incorporated in the latter two plasmids, respectively, are from pDIRECT_21C [2] and pTRAkt_HC [3]. Primers used for assembly are listed in Table S6. All constructs were transformed into *Agrobacterium fabrum* str. GV3101(pMP90).

Transient expression in *N. benthamiana*

Agrobacteria transformed with recombinant plasmids by freeze-thaw method were cultivated in LB medium (5 g/L yeast extract, 10 g/L NaCl, tryptone/peptone 10 g/L, pH 7.0) supplemented with

appropriate antibiotics at 28°C with shaking (180 rpm). After centrifugation (2000 x *g* for 10 min), the pellet was resuspended in infiltration buffer (10 mM MES-NaOH pH 5.6, 10 mM $MgSO_4$) to set the required OD_{600}. Cultures were then used for leaf-infiltration of 4–5-week-old *N. benthamiana* wild-type plants for expression of sgRNA constructs, and *N. benthamiana* partial FX-KO line NB14-29aT2 [4], and a β1,2-xylosyltransferase and α1,3-fucosyltransferase RNAi knockdown ΔXT/FT, respectively [5] and the full FX-KO line generated in this study for the expression of recombinant glyco-reporter proteins. Plants were cultivated at long-day settings (16 h light/ 8 h dark) at 25°C in 60% humidity.

Evaluation of gene editing by TIDE

For the evaluation of sgRNA efficiencies, the constructs were transiently expressed in *N. benthamiana* leaves. Samples were harvested at 5 dpi (days post infiltration) and gDNA was extracted. To extract genomic DNA, leave tissue was collected in a 2 mL tube and frozen in liquid nitrogen. Samples were ground to fine powder in a mixer mill with metal beads. Then 700 µL of extraction buffer (200 mM Tris-HCl pH 8.8, 250 mM NaCl, 25 mM EDTA, 0.5% SDS) was added and samples were mixed thoroughly. After centrifugation for 2 min at 16000 x *g*, 600 µL of the supernatant was collected and mixed with 600 µL of isopropanol. Then the samples were centrifuged for 20 min at maximum speed and the supernatant was discarded. Finally, the pellet was dried and resuspended in 50 µL of H_2O. The concentration of nucleic acid was measured with the NanoDrop spectrophotometer and ~100 ng of DNA was used as template for PCR (GoTaq Promega). Successful amplification of target regions was confirmed by agarose gel electrophoresis of a small reaction aliquot. PCR products were then treated with a mixture of thermolabile exonuclease I (ExoI, NEB) and shrimp alkaline phosphatase (rSAP, NEB) according to manufacturer's recommendations, before supplementing the inactivated reactions with a sequencing primer and submitting them for Sanger sequencing (Microsynth). Sequencing data was analyzed against wildtype control using the web interface of the TIDE application (Brinkman et al. 2014, http://shinyapps.datacurators.nl/tide/). Primers used to amplify the targeted gene regions are listed in Table S1.

Stable transformation of *Nicotiana benthamiana*

Seeds of the progenitor *N. benthamiana* KO line NB14-29aT2 [4] were sterilized in 70% ethanol (30 seconds) followed by 20 min shaking in 5% sodium hypochlorite containing 0.5% Tween-20. After five washes with sterile water, the seeds were distributed evenly on half-strength MS medium for germination. Agrobacterium cultures were spun down for 10 min at 3000 x *g*, resuspended in the liquid co-cultivation medium to OD_{600} 0.8 and used for transformation. One full plate (~50 seedlings) of 6 days old seedlings were collected by cutting off their roots. A toothpick was dipped into the Agrobacterium-containing

solution and used to wound the collected cotyledons to create an infiltration site. Cotyledons were then transferred to CCM plates abaxial side down and incubated at 25°C in the dark for three days [6]. Cotyledons were transferred to selection/shooting medium plates to select for transformants. Plates were kept in a growth chamber at 22°C with 16 h light/8 h dark photoperiod. Explants were sub-cultured every two weeks to maintain selective pressure. When shoots reached the size of around 5 mm, they were transferred to bud elongation medium, later to rooting medium and, once roots were developed, to soil (Figure S3). Media composition is listed in Table S7.

Screening for mutants

Genomic DNA was extracted from leaf discs and screened by PCR for the presence of Cas9 in T0 and its absence in T1 generation. Cas9 region of the transgene was amplified by PCR using primers Cas9_F and Cas9_R (594 bp amplicon). Primers for *N. benthamiana* catalase NbCat_F and NbCat_R (600 bp amplicon) were used as PCR positive control for the cases when Cas9 transgene was absent. To evaluate the extent of gene-editing in *NbFucT3*, *NbFucT4* and *NbFucT5,* respective gDNA regions including the target sites of interest were amplified separately by corresponding pairs of gene-specific primers FUT3-E1_F & FUT3-E1_R, FUT4-E1_F & FUT4-E1_R and FUT5_F1 & FUT5_R (Table S1), sequenced and analyzed by TIDE.

Expression of glycoproteins and enzymes for glycoengineering

Expression Vectors for human glycoproteins production have been described recently: monoclonal antibodies anti-CD20 IgG1 (rituximab [4]), anti-HER2 IgA2m1 (expected mass of heavy and light chain: ~55 and ~25 kDa) [7], anti-SARS-CoV2 IgM (heavy and light chain: ~70 and ~25 kDa) [8] and Fcab (25 kDa) [9]; human α1-antitrypsin (A1AT, ~50 kDa) [10] and Ig5FN1 (~35 kDa) module of human neural cell adhesion molecule (NCAM) [11]. The vector for β-1,4-galactosylation of a hybrid human galactosyltransferase with CTS region of a rat sialyltransferase regulated by octopine synthase (ocs) promotor/terminator was described previously in [12]. The used multigene vector pICH88266 for α2,6-sialylation of glycoproteins was described recently in (Izadi et al., 2023). Also, a hybrid fucosyltransferase with the catalytic domain of *Zea mays* core α1,3-fucosyltransferase and cytoplasmic tail, transmembrane domain and stem (CTS) region of *Arabidopsis thaliana* core α1,3-fucosyltransferase was used as described [13].

Extraction of total soluble proteins

Agroinfiltrated leaves (300 mg) were collected at 3 dpi and snap-frozen in liquid nitrogen. The tissue was ground to fine powder in a mixer mill (MM400, Retsch) with metal beads at 30 Hz for 2 min. To extract

total soluble proteins (TSP), extraction buffer (0.1 M Tris-HCl, 0.5 M NaCl, 1 mM EDTA, 40 mM ascorbic acid, pH 6.8-7.4) in a 1:2 w/v ratio was added. Non-soluble fraction was pelleted by centrifugation (13000 x *g* for 10 min) and the supernatant was used for affinity purification.

Antibody purification and isolation of apoplastic fluid from *N. benthamiana* leaves

Expressed IgG, IgA and IgM proteins were purified by affinity chromatography, using rProteinA Agarose Resin Fast Flow (Amicogen), CaptureSelect™ IgA Affinity Matrix (Thermo Scientific) and POROS™ CaptureSelect™ IgM Affinity Matrix (Thermo Scientific), respectively. Resin was equilibrated with phosphate-buffered saline (PBS, 137 mM NaCl, 2.7 mM KCl, 10 mM Na_2HPO_4, 1.8 mM KH_2PO_4, pH 7.4). Resin (30 µL) was incubated with the plant extract on a rotating mixer at 4°C for 90 min. After centrifugation (400 x *g*, 5 min), the resin was resuspended in PBS and loaded on a Micro Bio-Spin column (Bio-Rad). To recover the proteins from the resin, samples were eluted (100 mM glycine, pH 2.5) and immediately neutralized to pH 7.0 with counter buffer (1 M Tris-NaOH, pH 9.0). Protein concentrations were measured by NanoDrop spectrophotometer using protein-specific extinction coefficients.

Leaves containing Ig5FN1 and A1AT were vacuum-infiltrated with ice-cold extraction buffer (100 mM Tris–HCl, pH 7.5, 10 mM $MgCl_2$, 2 mM EDTA). Any excess buffer was removed and apoplastic fluid was collected in a 50 mL Falcon tube with inserted mesh by low-speed centrifugation (800 x *g*, 10 min at 4°C).

SDS-PAGE

Approximately 2-4 µg of protein were reduced in Laemmli buffer at 95°C for 5 min and separated by SDS-PAGE in a 12% AA gel. For visualization Coomassie Brilliant Blue (R 250) staining was used.

Total endogenous N-glycan analysis by MALDI-TOF

One gram of leaf tissue was homogenized with 5% formic acid, and digested by pepsin (37°C, overnight). Purification was done by ion exchange chromatography (Dowex-Gel 50Wx2-400) and by gel filtration (Sephadex G25). Subsequently, the samples were digested with PNGase A to release glycans followed by purification via ion exchange chromatography (Dowex-Gel 50Wx2-400), and by solid phase extraction (Phenomenex Strata C18-E). Measurement of the total endogenous N-glycans was done by MALDI-TOF MS [14].

In-gel and in-solution digestion

Fractions containing the glycosites of IgG, Fcab, A1AT, Ig5FN1 samples were cut from SDS-PAGE gels and digested in-gel. For that, the gel fractions were washed two times with 50% acetonitrile (50 µL, 15 min) and once with 100% acetonitrile (50 µL, 30 sec). Then, samples were incubated in 0.1 M NH_4HCO_3 (30 µL, room temperature (RT), 5 min) and 100 % acetonitrile (30 µL, 15 min). A vacuum concentrator (SpeedVac, 15 min) was used to dry the samples. Cysteines were reduced (50 µL 10 mM DTT for 45 min at 56°C), and S-alkylated (50 µL 55 mM iodoacetamide), 30 min in the dark at RT). After repeated washing and drying as described before, the samples were digested by trypsin (3.33 µL, Promega, sequencing grade 0.1 µg/µL) in 25 mM NH_4HCO_3 (70 µL, ~18 h at 37°C). Only Ig5FN1 was digested by trypsin and GluC (Promega) following manufacturer's protocol. Then, peptides were extracted by incubating gel pieces in 25 mM NH_4HCO_3 (30 µL) and followed by 100 % acetonitrile (30 µL) for 15 min with shaking in a thermomixer. The extraction was repeated twice and the residue was extracted in 5% formic acid (30 µL), after which the combined extracts were dried in a vacuum concentrator.

For in-solution digestion of IgA and IgM, approximately 20-25 µg of purified protein was used. The samples were mixed with NH_4HCO_3 (30 µL of 0.1 M) and cysteines were reduced with 15 mM DTT (30 µL) and S-alkylated as described above. The proteins were then precipitated by ice-cold acetone (500 µL, 60 min, -20°C). After centrifugation (13000 x *g*, 7 min, 4°C), removal of supernatant, drying in a vacuum concentrator and dissolving of the pellet in 0.1 M NH_4HCO_3 (30 µL), the sample was digested with trypsin (5 µL, 37°C, 18 h, Promega, sequencing grade 0.1 µg/µL).

LC-ESI-MS based glycan analysis

Digested peptide mixtures were analyzed for their site-specific N-glycosylation profile by reversed-phase liquid chromatography-electrospray ionization mass spectrometry (LC-ESI-MS). IgA2m1 and Fcab were analyzed using a Dionex Ultimate 3000 system directly linked to a Q-TOF instrument (maXis 4G ETD, Bruker) equipped with the standard ESI source in the positive ion DDA mode (= switching to MS/MS mode for eluting peaks). MS-scans were recorded (range: 150–2200 m/z) and the six highest peaks were selected for fragmentation. Instrument calibration was performed using ESI calibration mixture (Agilent). For peptide separation, a Thermo BioBasic C18 separation column (5 µm particle size, 150 × 0.360 mm) was used. A gradient from 97% solvent A and 3% solvent B (Solvent A: 65 mM ammonium formate buffer, B: 100% acetonitrile) to 32% B in 45 min was applied, followed by a 15 min gradient from 32% B to 75% B, at a flow rate of 6 µL/minute. MS-spectra as well as the acquired MS/MS-scans of dominant precursor peaks were manually analyzed and annotated using Data Analysis 4.0 (Bruker). Glycopeptides were quantified based on signal intensity (peak height) of the highest isotope peak of each glycopeptide, assuming the

isotopic pattern is the same between the analyzed ions. IgG1, IgM A1AT, Ig5FN1 were analyzed using a Thermo Orbitrap Exploris 480. The possible glycopeptides were identified as sets of peaks consisting of the peptide moiety and the attached N-glycan varying in the number of HexNAc units, hexose, deoxyhexose, and pentose residues. Manual glycopeptide searches were performed using FreeStyle 1.8 (Thermo), deconvolution was done using the extract function. The peak heights roughly reflect the molar ratios of the glycoforms. Glycan nomenclature is according to the Consortium for Functional Glycomics (www.functionalglycomics.org). Glycan symbols used are according to the Symbol Nomenclature for Glycans (SNFG) (https://www.ncbi.nlm.nih.gov/glycans/snfg.html).

Antigen-binding ELISA

IgG1 (rituximab) ELISA: a 20-mer peptide (P20) of the extracellular loop of human CD20 was used as the antigen [15]. 1 µg/mL of P20 (diluted in PBS buffer, pH 7.4) was coated (50 µL/well) to 96 well microplates (MicroWell™ MaxiSorp™ Merck SA M9410-1CS) overnight at 4°C, then saturated by incubation 100 µL/well with 3% fat free milk powder, dissolved in PBS-T (PBS with 0.05% Tween 20) for 1.5 h at RT. Solutions of rituximab was diluted in blocking solution and applied to the coated plates in two-fold serial dilutions starting from 1000 µg/mL were then added (50 µL/well) and incubated for 2 h at RT to obtain calibration curves. Peroxidase-conjugated goat anti-human gamma chain antibody (Merck SA I3382) was added at a dilution of 1:5000 and the plates were incubated for a further hour at RT. The substrate 3,3',5,5'-tetramethylbenzidine (Merk SA) was added (50 µL/well) and plates were incubated for ~10 min. The reaction was stopped with 2 M H_2SO_4 and absorbance (λ = 450 nm) with reference to 620 nm was measured with an ELISA reader (Tecan Spark® spectrophotometer). All samples were analyzed at least in two technical replicates. EC50 values were calculated by non-linear regression of the blank-corrected data points based on a four-parametric log model with GraphPad Prism (version 9).

FcγR binding by flow cytometry

FcγRIIIa (CD16A; F158 allotype) expressing TZM-bl cells were used to assess the binding affinity of rituximab purified by Protein A chromatography. Wild-type TZM-bl cells were included as a negative control to account for unspecific antibody binding. Cells were detached with Accutase, washed, and seeded into 96-well plates at $1x10^5$ cells per well. Serially diluted (1:4) antibodies were added at a starting concentration of 100 µg/mL and incubated for 45 min at 4°C). Cells were washed twice with PBS and resuspended in a 1:200 dilution of a PE-labeled Fab fragment goat anti-human IgG F(ab')2 conjugate (Jackson ImmunoResearch). After 30 min cells were washed twice and fixed in a 4% paraformaldehyde solution. Cells were then analyzed by flow cytometry on a NovoCyte flow cytometer (ACEA). The binding

curves were generated by plotting the mean fluorescence intensity (MFI) of PE-positive cells indicating receptor binding as a function of Ab concentration. Unspecific binding to wild-type cells was subtracted from binding to FcγRIIIa-expressing cells. Each antibody concentration was run in duplicate. Binding experiments were repeated three times and visualized as median binding without SD because of the small number of replicates.

Supplementary Tables

Table S1 Primers used for screening.

Primer name	5‘ -> 3‘ sequence	Primer specificity
Cas9_F	CTGACGTCGATAAGTTGTTCA	*SpCas9*
Cas9_R	TGATGAACTTGTAGAACTCCT	
nptII_F	TTGTCACTGAAGCGGGAAGG	*nptII*
nptII_R	TAAAGCACGAGGAAGCGGTC	
NbCat1	CATTCGCGGTTTTGCTGTC	*N.benth.* Catalase
NbCat2	TGGTGGCGTGGCTATGATTTGTA	
FUT3-E1_F	AACTGTCCCACCAAATGAAG	Exon 1 of *NbFucT3*
FUT3-E1_R	TTACCCTGCATTTCTGCG	
FUT4-E1_F	AACTGTCCCACCAAATGAAT	Exon 1 of *NbFucT4*
FUT4-E1_R	AGAACTAACACTAGAGGACAGTAG	
FUT3-E3_F	GACACAATAACCTTAGGATCATGG	Exon 3 of *NbFucT3*
FUT3-E3_R	GAAATTGAGAACAAGATGACAAGTG	
FUT4-E3_F	AGACACAATAACCTTAGGATCATGT	Exon 3 of *NbFucT4*
FUT4-E3_R	AGATGTCTGCTTTCAACTATATCC	
FUT3-E4_F	CATGCATGTGGTTAAACTTTACATTATATG	Exon 4 of *NbFucT3*
FUT3-E4_R	ATAGAGCAGAGCATATTTGTGAAC	
FUT4-E4_F	CGTGCATGCATAAAAAATGTCTG	Exon 4 of *NbFucT4*
FUT4-E4_R	ATAGAGCAGAGCATATTTGTACGT	
FUT5_F1	GGTGTTGGGTCATCATCC	*NbFucT5*
FUT5_R	GAATTTTTCGGTGTCCTCATTG	

Table S2 Genes targeted in the present study.

Gene	Accession		
	Sol Genomics Network (https://solgenomics.net/)	Queensland University of Technology (http://benthgenome.qut.edu.au/)	NbSC Web Apollo LAB3.60 (https://www.nbenth.com/)
NbFucT3	Niben101Scf05494	Nbv0.5scaffold1070	NbL09g22590.1
NbFucT4	Niben101Scf17626	Nbv0.5scaffold8260	NbL08g16470.1
NbFucT5	Niben101Scf05447	Nbv0.5scaffold1460	NbL03g05760.1

Table S3 Spacer sequences of RNA guides targeting *N. benthamiana* FucT genes selected for assessment of their actual on-target transient activity in leaf cells of NB14-29aT2 plants. Only one sgRNA (G1.3) targeting exon 1 proved to effectively induce mutations in the transient assay. Interestingly, G1.3 was also ranked with the highest efficacy score by CRISPRater.

sgRNA ID	target/spacer sequence (5' -> 3')	Efficacy score by CRISPRater	Transient activity in experiment	Location of the main target
G1.1	CCCAAAAGAAATGGTCCAAT	0.44 LOW	-	Exon 1 of *NbFucT3* and *NbFucT4*
G1.2	ACCAGTTTACGACGTCGTCT	0.62 MEDIUM	-	
G1.3	AGGCTGGGTTGGGTGTGTTG	0.88 HIGH	+	
G1.4	CTGTGAGGAGTGGTTGGAAA	0.67 MEDIUM	-	
G1.5	AAAGATCCAATTTTTGTTCA	0.67 MEDIUM	-	
G3.1	GCTGCTTTTATTTCTAATTG	0.72 MEDIUM	-	Exon 3 of *NbFucT3* and *NbFucT4*
G3.2	AGGCTCTTGAAGTCCTTGAA	0.69 MEDIUM	-	
G3.3	TGGCAGTTGTCATCGTAACC	0.65 MEDIUM	-	
G4.1	CGCTTTTGAGAATTCTAATG	0.69 MEDIUM	-	Exon 4 of *NbFucT3* and *NbFucT4*
G4.2	TGTTCTCAATTTCATGGCAG	0.69 MEDIUM	-	
G4.3	TTTCATGGCAGTGGACAAAG	0.58 MEDIUM	-	

Table S4 Editing efficiency of G1.3 upon transient expression. (A) sgRNA (G1.3) targeting exon 1 proved to effectively induce mutations in the transient assay, resulting in about 18% and 14% of gDNA molecules with *NbFucT3* and *NbFucT4*, respectively, to contain indels at the expected site. **(B)** We found that transient editing efficiency of G1.3 was considerably improved when the respective sgRNA transcript was expressed alone from the enhanced 35S promoter compared to when it was a part of the multiplexed cluster driven by CmYLCV promoter. Data from the transient assay with a construct pBG04 featuring G1.3 in this arrangement showed the overall indel incidence increased to approximately 43% for *NbFucT3* and 36% for *NbFucT4*.

		**(A)** pDV15		**(B)** pBG04	
**Spacer sequence**	**Target**	**Indel type detected**	**Indel frequency, %**	**Indel type detected**	**Indel frequency, %**
AGGCTGGGTTGGGTGTGTTG	*NbFucT3* (exon 1)	-1	18	+1<br>-1	6<br>38
	NbFucT4 (exon 1)	-1	14	-1<br>-3<br>+1	32<br>3<br>5

Table S5 **Polypeptide products (theoretical for *NbfucT5*) of knocked out genes in NbBG04-4-18-14 (NbXF-KO).**

Mutated gene	AA sequence of the polypeptide product corresponding to CDS of the mutated gene
NbfucT3	MATVIPIQRLPRFEGVGSSSPTNAPQKKWSNWLPLVVGLVVLVEIAFLGRLDMAEKANLVNSWTDSFYQFTTSSWSTSKVEINEAGLGV*
NbfucT4	MATVIPIQRIPRFEGVGSLSPTNVPQKKWSNWLPLVVALVVIVEIAFLGRLDMAEKANLVNSWTDSFYQFTTSSWSTSNVEINEAGLGFEE*
NbfucT5	METVIPIQRIPRFEGVGSSSPTNVPQKKWSNWLPLIVALVVIVEIAFLGRLEMAEKANLVNSWTDSFYQFTTSFWSTSKVEINEAGLGVFEE*

Table S6 **Primers used for assembly of sgRNA cassettes**.

Primer name	5‘ -> 3‘ sequence
NbFUT-G1.1_MTR	ACGTCTCAATTTCTTTTGGGTGCACCAGCCGGGAATC
NbFUT-G1.1_MTF	ACGTCTCTAAATGGTCCAATGTTTCAGAGCTATGCTGG
NbFUT-G1.2_MTR	ACGTCTCATCGTAAACTGGTTGCACCAGCCGGGAATC
NbFUT-G1.2_MTF	ACGTCTCTACGACGTCGTCTGTTTCAGAGCTATGCTGG
NbFUT-G1.4_MTR	ACGTCTCACACTCCTCACAGTGCACCAGCCGGGAATC
NbFUT-G1.4_MTF	ACGTCTCTAGTGGTTGGAAAGTTTCAGAGCTATGCTGG
NbFUT-G3.2_MTR	ACGTCTCACTTCAAGAGCCTTGCACCAGCCGGGAATC
NbFUT-G3.2_MTF	ACGTCTCTGAAGTCCTTGAAGTTTCAGAGCTATGCTGG
NbFUT-G4.3_MTR	ACGTCTCAACTGCCATGAAATGCACCAGCCGGGAATC
NbFUT-G4.3_MTF	ACGTCTCTCAGTGGACAAAGGTTTCAGAGCTATGCTGG
NbFUT-G1.3_MTR	ACGTCTCACCAACCCAGCCTTGCACCAGCCGGGAATC
NbFUT-G1.3_MTF	ACGTCTCTTTGGGTGTGTTGGTTTCAGAGCTATGCTGG
NbFUT-G3.1_MTR	ACGTCTCAAATAAAAGCAGCTGCACCAGCCGGGAATC
NbFUT-G3.1_MTF	ACGTCTCTTATTTCTAATTGGTTTCAGAGCTATGCTGG
NbFUT-G3.3_MTR	ACGTCTCATGACAACTGCCATGCACCAGCCGGGAATC
NbFUT-G3.3_MTF	ACGTCTCTGTCATCGTAACCGTTTCAGAGCTATGCTGG
NbFUT-G4.2_MTR	ACGTCTCAAAATTGAGAACATGCACCAGCCGGGAATC
NbFUT-G4.2_MTF	ACGTCTCTATTTCATGGCAGGTTTCAGAGCTATGCTGG
NbFUT-G1.5_MTR	ACGTCTCAATTGGATCTTTTGCACCAGCCGGGAATC
NbFUT-G1.5_MTF	ACGTCTCTCAATTTTTGTTCAGTTTCAGAGCTATGCTGG
SapI-UGR3_F	TGCTCTTCCATGTCGACGCCCGGTCCAAAGACC
SapI-UGR_F	TGCTCTTCCATGTCGAGCTGGCAGACATACT
SapI-UGR_R	TGCTCTTCATGCGATCCACTTGCATAGCGAGTCAG

Table S7 **Media used for plant tissue culture.**

Media	Components
Seed germination medium	½ MS basal salts, 10 g/L Sucrose, 2.5 g/L Phytagel, pH 5.7
Transformation/co-cultivation medium	1x MS basal salts, 30 g/L Sucrose, 1x Gamborg vitamins, 2 mg/L benzylaminopurine hydrochloride, 100 µM acetosyringone, 2.5 g/L Phytagel, pH 5.7
Selection/shooting medium	1x MS basal salts, 30 g/L sucrose, 1x Gamborg vitamins, 2 mg/L benzylaminopurine hydrochloride, 100 mg/L Kanamycin sulfate, 100 mg/L timentin, 2.5 g/L Phytagel, pH 5.7
Bud elongation medium	1x MS basal salts, 30 g/L sucrose, 1x MS vitamins, 0.1 mg/L benzylaminopurine hydrochloride, 100 mg/L Kanamycin sulfate, 100 mg/L timentin, 2.5 g/L Phytagel, pH 5.7
Rooting medium	1x MS basal salts, 30 g/L sucrose, 1x MS vitamins, 100 mg/L Kanamycin sulfate, 2.5 g/L Phytagel, pH 5.7

Table S8 **Glycopeptides derived from enzymatic digest of glyco-reporter proteins used in this study.** Glycosites are indicated in bold and underlined.

Protein	Glycopeptides after enzymatic digest	Glycosite	Monoisotopic mass (Da)
IgG-Fc	EEQY<u>**NST**</u>YR	N297	1189.5120
IgM	GS1: YK<u>**NNS**</u>DISSTR	N46	1284.6179
	GS2: GLTFQQ<u>**NAS**</u>SMCVPDQDTAIR	N209	2339.0754
	GS3: THT<u>**NIS**</u>E	N272	801.3737
	GS4: SHP<u>**NAT**</u>FSAVGE	N279	1216.5593
	GS5: STGKPTLY<u>**NVS**</u>LVMSDTAGTCY	N440	2365.105
IgA2m1	GS1: SVTWSESGQ<u>**NVT**</u>AR	N47	1521.7292
	GS2: LSLHRPALEDLLLGSEA<u>**NLT**</u>CTLTGLR	N131	2963.5982
	GS3: TPLTA<u>**NIT**</u>K	N205	9585.5670
	GS4: LAGKPTHV<u>**NVS**</u>VVMAEVDGTCY	N327	2347.1420
Ig5FN1	GS4: GNQV<u>**NIT**</u>CE	N20*	1034.4571
	GS5: DGQLLPSS<u>**NYS**</u>NIK	N46*	1535.7700
	GS6: NDFGNY<u>**NCT**</u>AVNR	N75*	1544.6546
A1AT	GS1: QLAHQS<u>**NST**</u>NIFFSPVSIATAFAMLSLGTK	N46	3181.6350
	GS2: ADTHDEILEGLNF<u>**NLT**</u>EIPEAQIHEGFQELLR	N83	3691.8238
	GS3: YLG<u>**NAT**</u>AIFFLPDEGK	N247	1755.8952

*AA Nr of the mature peptide of Ig5FN1 construct GenBank database (accession no. KU052570).

Table S9 **Relative N-glycan distribution of total soluble proteins (TSP) and on distinct glycosylation sites (GS) of purified glycoproteins.**

Sample	Expression host	MM	MMF	GnM	GnMF	GnGn	GnGnF	AA	AM	AMan4	AMan5	Gn(FA)	AGn	NaM	NaGn	NaA	NaNa	Mannosidic	others >5%	Sum
TSP plant 1	NB14-29aT1	7	22	5	6	9	39	0	0	0	0	0	0	0	0	0	0	12	0	100
TSP plant 2	NB14-29aT2	5	18	6	11	9	39	0	0	0	0	0	0	0	0	0	0	12	0	100
TSP plant 3	NB14-29aT3	5	21	5	10	9	38	0	0	0	0	0	0	0	0	0	0	13	0	100
TSP plant 4	NB14-29aT4	6	21	5	9	8	37	0	0	0	0	0	0	0	0	0	0	10	4	100
TSP	average	6	20	5	9	9	38	0	0	0	0	0	0	0	0	0	0	12	1	100
TSP plant 1	NbXF-KO	24	0	20	0	41	0	0	0	0	0	0	0	0	0	0	0	13	1	100
TSP plant 2	NbXF-KO	23	0	20	0	41	0	0	0	0	0	0	0	0	0	0	0	14	2	100
TSP plant 3	NbXF-KO	23	0	21	0	41	0	0	0	0	0	0	0	0	0	0	0	13	2	100
TSP plant 4	NbXF-KO	25	0	21	0	43	0	0	0	0	0	0	0	0	0	0	0	11	1	100
TSP plant 5	NbXF-KO	26	0	21	0	38	0	0	0	0	0	0	0	0	0	0	0	13	2	100
TSP plant 6	NbXF-KO	28	0	19	0	39	0	0	0	0	0	0	0	0	0	0	0	12	1	100
TSP	average	25	0	20	0	40	0	0	0	0	0	0	0	0	0	0	0	13	2	100
IgG1 plant 1	NbXF-KO	1	0	10	0	82	0	0	0	0	0	0	0	0	0	0	0	7	0	100
IgG1 plant 2	NbXF-KO	1	0	9	0	83	0	0	0	0	0	0	0	0	0	0	0	7	0	100
IgG1 plant 3	NbXF-KO	1	0	10	0	82	0	0	0	0	0	0	0	0	0	0	0	8	0	100
IgG1 plant 4	NbXF-KO	1	0	9	0	84	0	0	0	0	0	0	0	0	0	0	0	7	0	100
IgG1	average	1	0	9	0	83	0	0	0	0	0	0	0	0	0	0	0	7	0	100
A1AT GS2	NbXF-KO	34	0	12	0	53	0	0	0	0	0	0	0	0	0	0	0	0	0	100
A1AT GS3	NbXF-KO	35	0	19	0	44	0	0	0	0	0	0	0	0	0	0	0	0	3	100
Ig5FN1 GS4	NbXF-KO	17	0	64	0	13	0	0	0	0	0	0	0	0	0	0	0	0	5	100
Ig5FN1 GS5	NbXF-KO	10	0	20	0	65	0	0	0	0	0	5	0	0	0	0	0	0	0	100
Ig5FN1 GS6	NbXF-KO	3	0	14	0	81	0	0	0	0	0	0	0	0	0	0	0	0	1	100
IgM GS1	NbXF-KO	1	0	81	0	15	0	0	0	0	0	0	0	0	0	0	0	2	1	100
IgM GS2	NbXF-KO	1	0	11	0	85	0	0	0	0	0	0	0	0	0	0	0	2	1	100
IgM GS3	NbXF-KO	0	0	3	0	96	0	0	0	0	0	0	0	0	0	0	0	2		100
IgM GS4	NbXF-KO	1	0	0	0	0	0	0	0	0	0	0	0	0	0	0	0	99	0	100
IgM GS5	NbXF-KO	0	0	1	0	6	0	0	0	0	0	0	0	0	0	0	0	89	3	100
IgA GS1	NbXF-KO	0	0	23	0	65	0	0	0	0	0	0	0	0	0	0	0	12	0	100
IgA GS2	NbXF-KO	8	0	32	0	19	0	0	0	0	0	0	0	0	0	0	0	41	0	100
IgA GS3	NbXF-KO	23	0	31	0	33	0	0	0	0	0	0	0	0	0	0	0	12	0	100
IgA GS4	NbXF-KO	0	0	5	0	43	0	0	0	0	0	0	0	0	0	0	0	52	0	100
IgG1 +ST-GalT	NbXF-KO	3	0	12	0	3	0	35	29	8	4	0	7	0	0	0	0	0	0	100
A1AT+PICH GS2	NbXF-KO	1	0	2	0	7	0	0	0	0	0	0	0	15	5	2	68	0	0	100
A1AT +PICH GS3	NbXF-KO	9	0	4	0	7	0	0	0	0	0	0	0	20	3	3	55	0	0	100
Ig5FN1 +PICH GS4	NbXF-KO	12	0	21	0	9	0	0	0	0	0	0	0	38	2	2	13	0	4	100
Ig5FN1 +PICH GS5	NbXF-KO	13	0	15	0	35	0	0	0	0	0	0	0	0	7	1	21	0	8	100
Ig5FN1 +PICH GS6	NbXF-KO	6	0	12	0	39	0	0	0	0	0	0	0	13	3	2	24	0	0	100

Table S10 **Relative N-glycan distribution on distinct glycosylation sites (GS) of purified glycoproteins.** The data for Fcab represents the average of six different plants.

Sample	Expression host	MM	MMX	MMF	GnM	GnMX	GnMF	GnMXF	GnGn	GnGnF	NaNa	NaMF	NaAF	NaNaF	Mannosidic	Others	Sum
Fcab	NB14-29aT1	0	0	9	0	0	0	0	75	16	0	0	0	0	0	0	100
IgM GS1	ΔXT/FT	0	0	0	51	0	8	0	17	13	0	0	0	0	0	10	100
IgM GS2	ΔXT/FT	0	0	0	8	0	0	0	67	18	0	0	0	0	0	8	100
IgM GS3	ΔXT/FT	0	0	0	4	0	0	0	83	11	0	0	0	0	0	2	100
IgM GS4	ΔXT/FT	0	0	0	0	0	0	0	0	0	0	0	0	0	97	3	100
IgM GS5	ΔXT/FT	0	0	0	0	0	0	0	0	0	0	0	0	0	97	3	100
IgG1 +FucT +PICH	NbXF-KO	0	0	0	0	0	0	0	0	6	3	11	3	62	11	4	100

Supplementary Figures

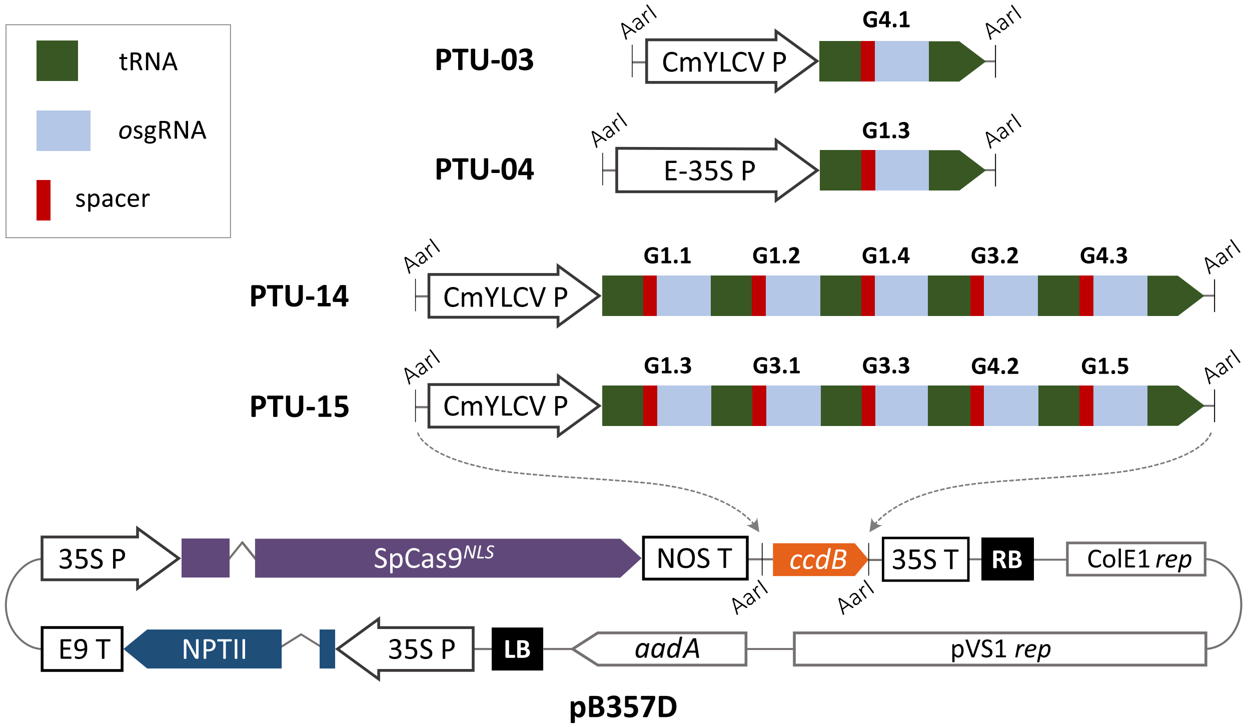

Figure S1 Illustration of CRISPR/Cas9 vector constructs used in this study. The binary vectors pBG03, pBG04, pDV14 and pDV15 were constructed by replacing ccdB gene in the destination plasmid pB357D with polycistronic transcription units PTU-03, PTU-04, PTU-14 and PTU-15, respectively. LB: left border repeat from octopine Ach5 T-DNA; 35S P: cauliflower mosaic virus 35S promoter, NPTII: neomycin phosphotransferase II containing IV2 intron from the potato ST-LS1 gene; E9 T: *Pisum sativum* ribulose-1,5-bisphospate carboxylase small subunit gene terminator; SpCas9^{NLS}: *Streptococcus pyogenes* CRISPR associated protein 9 with nuclear localization signals (SV40 at N- and nucleoplasmin at C-terminus) and rbcS intron from the *Pisum sativum* ribulose-1,5-bisphospate carboxylase small subunit gene; NOS T: nopaline synthase terminator; CmYLCV P: Cestrum yellow leaf curling virus promoter; E-35S P – enhanced CaMV 35S promoter; *o*sgRNA: optimized single guide RNA scaffold [16]; tRNA: *Arabidopsis thaliana* tRNA-Gly; *ccdB*: bacterial gene encoding DNA gyrase inhibitor; 35S T: polyadenylation signal/terminator from cauliflower mosaic virus; RB: right border repeat from nopaline C58 T-DNA; *aadA*: bacterial gene for aminoglycoside adenylyltransferase; pVS1 *rep*: replicon from *Pseudomonas* plasmid pVS1, ColE1 *rep*: high-copy-number bacterial replicon from ColE1 plasmid.

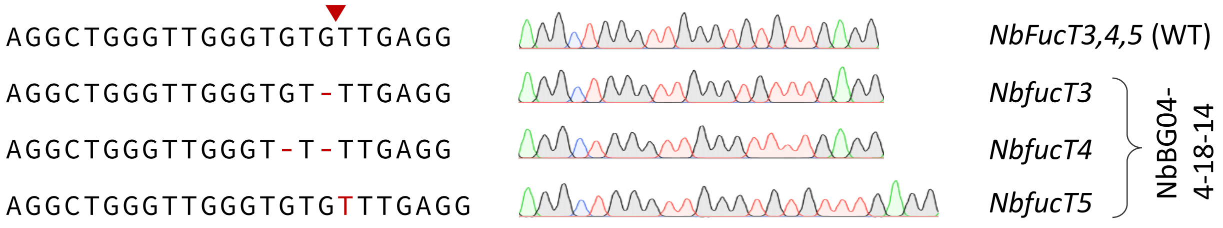

Figure S2 DNA sequencing results demonstrating homozygous mutations in FucT genes of the *N. benthamiana* line NbBG04-4-18-14 (NbXF-KO). Position of the double strand break induced by SpCas9/G1.3 complex is represented with a red triangle.

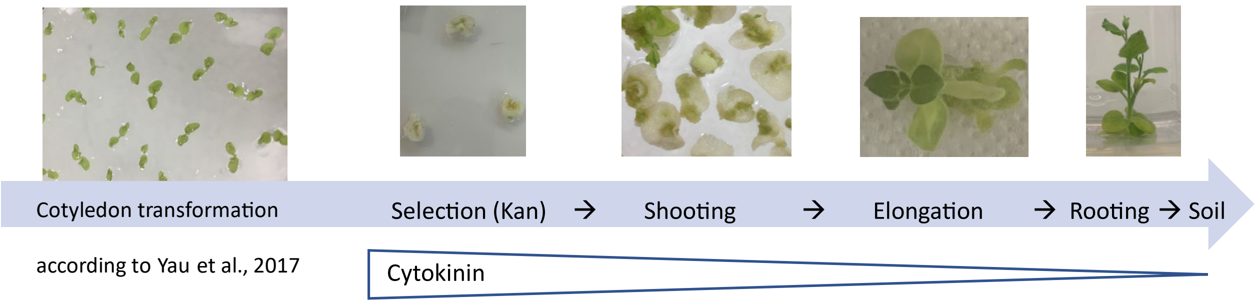

Figure S3 Tissue culture workflow. Cotyledon transformation was performed as described in [6]. Transformed Cotyledons were placed on selection media (kanamycin), followed by shooting and elongation under decreasing cytokinin concentration. Regenerants were transferred to rooting media, and after developing sufficient amounts of roots they were transferred to soil.

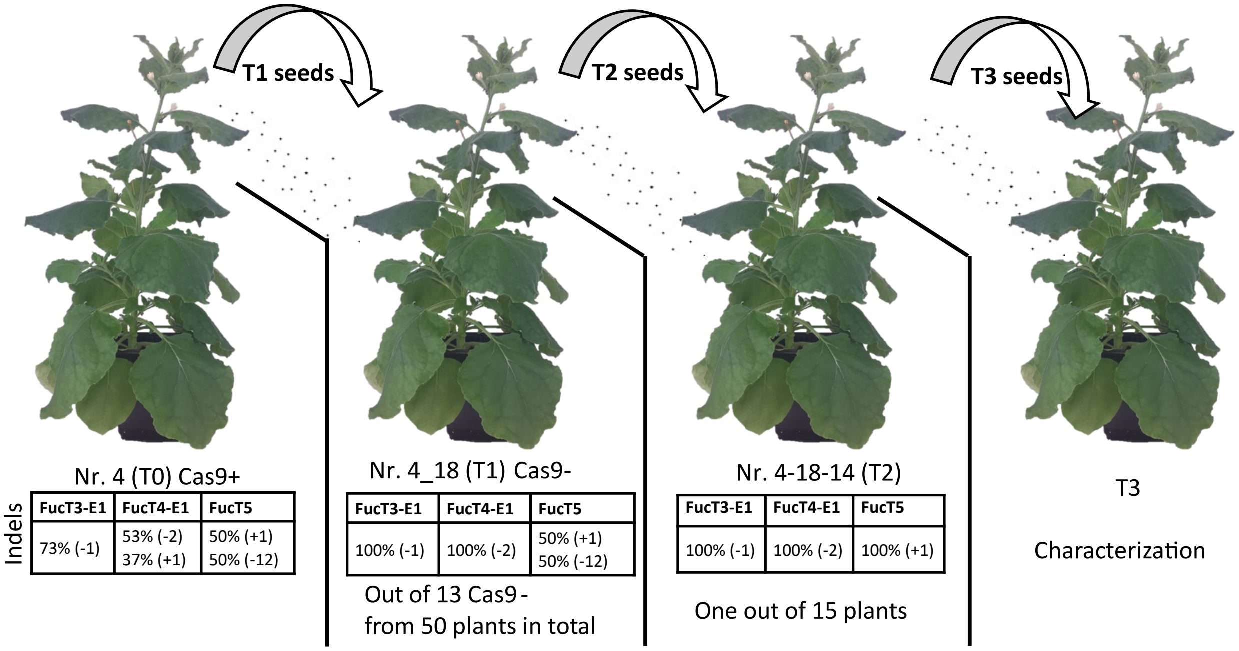

Figure S4 Overview of screening and indel frequencies over 3 generations. Transformant Nr. 4 (T0) had indels in all fucosyltransferases. In T1 13 Cas9-negative plants were found out of 50 tested, and one plant out of those had indel spectrum corresponding to homozygous state of the frame-shifting mutations in *FucT3* and *FucT3*, while mutations in *FucT5* were still biallelic (+1/-12). In T2 one plant out of 15 could be identified as homozygous for the desired mutation (+1) in *FucT5*. In T3 several plants were subjected to N-glycan characterization.

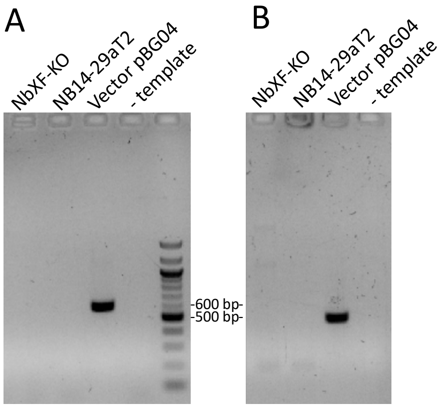

Figure S5 Gel electrophoresis of PCR products obtained with Cas9- and *nptII*-specific primers of NbXF-KO line. Expected size of Cas9 amplicon: 597 bp and of *nptII* amplicon: 484bp.

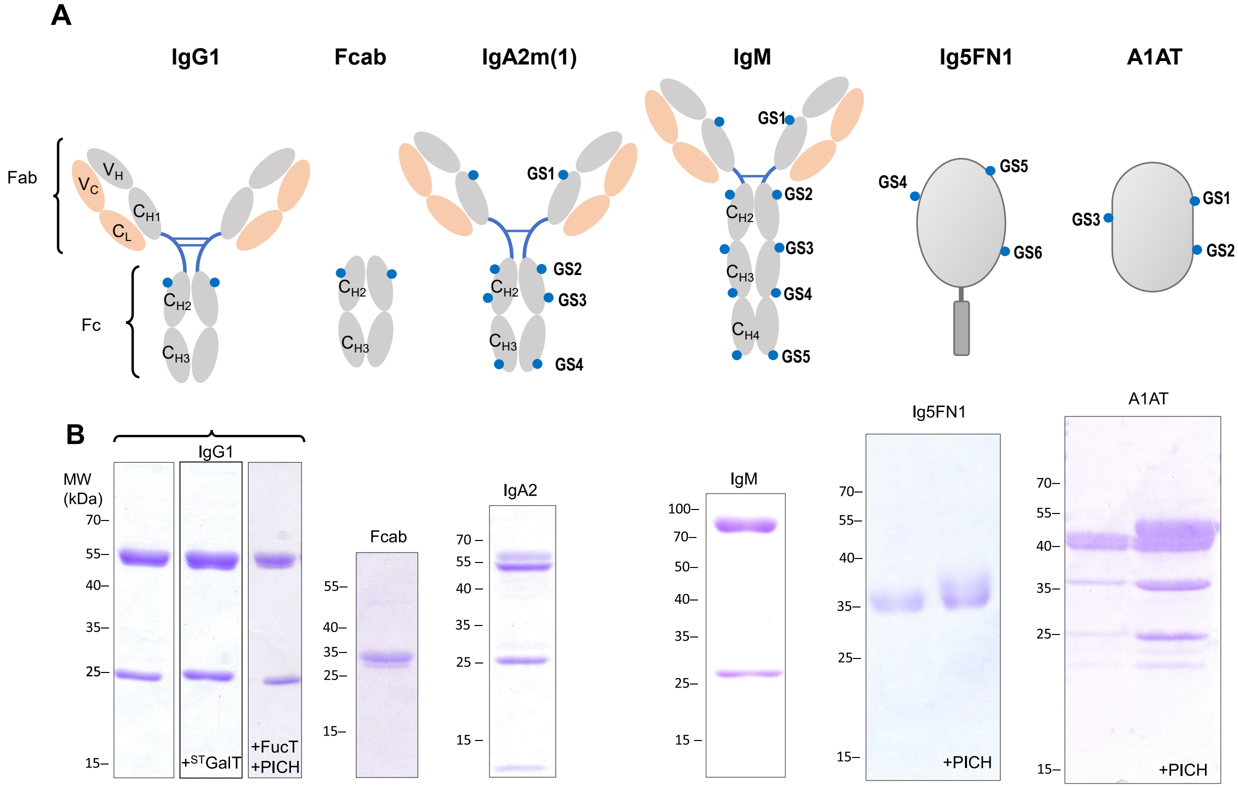

Figure S6 Illustration of reporter proteins. (a) Schematic presentation of reporter glycoproteins. Blue dots indicate N-glycosylation sites (GS, ascending numbering from N-C-terminus); Fcab: IgG1 Fc-fragment; CH, VH: constant and variable heavy chain; CL, VL: constant and variable light chain; Fab: fragment antigen binding; Ig5FN1: module of human neural cell adhesion molecule (NCAM, GS numbering resembles GSs 4, 5 and 6 of the full NCAM protein), A1AT: human α1-antitrypsin. IgM: Immunoglobulin M; IgA: Immunoglobulin A2m; GS: glycosite **(b)** SDS-PAGE (CBB stained) of purified recombinant proteins. Side bars represent molecular mass (in kDa), ST-GalT: modified β1,4 galactosyltransferase (Kallolimath et al., 2018); PICH: multigene vector for sialylation [17], FucT: hybrid Zea mais α1,3-fucosyltransferase [13].

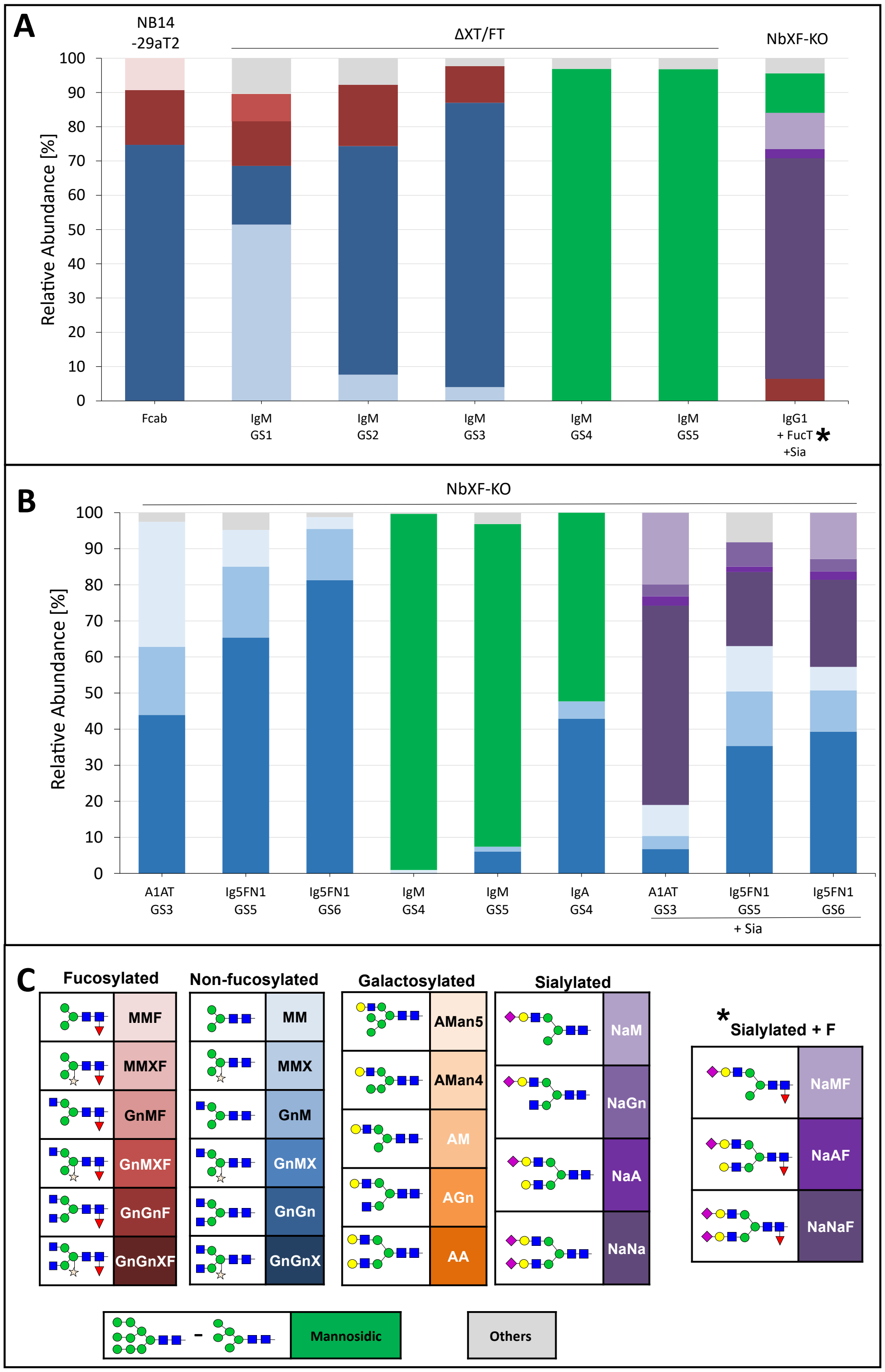

Figure S7 (A) N-glycan composition of reporter proteins expressed in NB14-29aT2, ΔXT/FT and NbXF-KO line. **(B)** reporter proteins expressed in NbXF-KO: glycosites (GS) not shown in Figure 2A; +Sia: coexpression of pICH 88266 [17]; FucT: hybrid Zea mais α1,3-fucosyltransferase [13]. * IgG1+FucT+Sia: NaNaF and NaNa are displayed combined. For detailed information see Figure S8, Tables S8, S9 and S10. **(C)** Schematic presentation of glycoforms according to Consortium for Functional Glycomics (www.functionalglycomics.org).

A

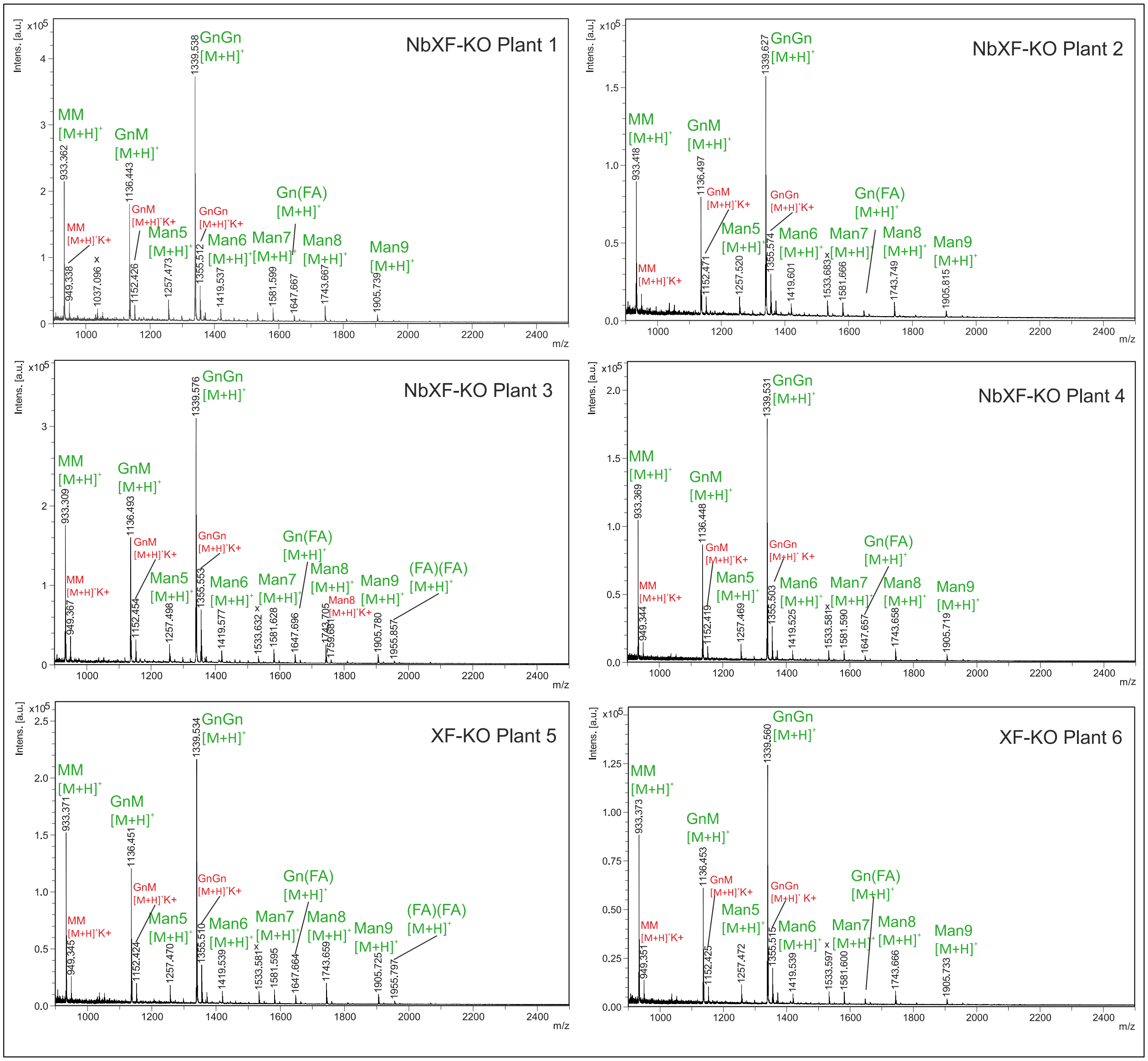
NbXF-KO Plant 1
Intens. [a.u.]
x10^5
GnGn [M+H]+
MM [M+H]+
GnM [M+H]+
MM [M+H]+K+
GnM [M+H]+K+
GnGn [M+H]+K+
Man5 [M+H]+
Man6 [M+H]+
Man7 [M+H]+
Gn(FA) [M+H]+
Man8 [M+H]+
Man9 [M+H]+
933.362
949.338
1037.096
1136.443
1152.426
1257.473
1339.538
1355.512
1419.537
1581.599
1647.667
1743.667
1905.739
m/z
NbXF-KO Plant 2
933.418
1136.497
1152.471
1257.520
1339.627
1355.574
1419.601
1533.683
1581.666
1743.749
1905.815
NbXF-KO Plant 3
(FA)(FA) [M+H]+
Man8 [M+H]+K+
933.309
949.367
1136.493
1152.454
1257.498
1339.576
1355.553
1419.577
1533.632
1581.628
1647.696
1743.705
1759.681
1905.780
1955.857
NbXF-KO Plant 4
933.369
949.344
1136.448
1152.419
1257.469
1339.531
1355.503
1419.525
1533.581
1581.590
1647.657
1743.658
1905.719
XF-KO Plant 5
933.371
949.345
1136.451
1152.424
1257.470
1339.534
1355.510
1419.539
1533.581
1581.595
1647.664
1743.659
1905.725
1955.797
XF-KO Plant 6
933.373
949.351
1136.453
1152.425
1257.472
1339.560
1355.515
1419.539
1533.597
1581.600
1743.666
1905.733

B

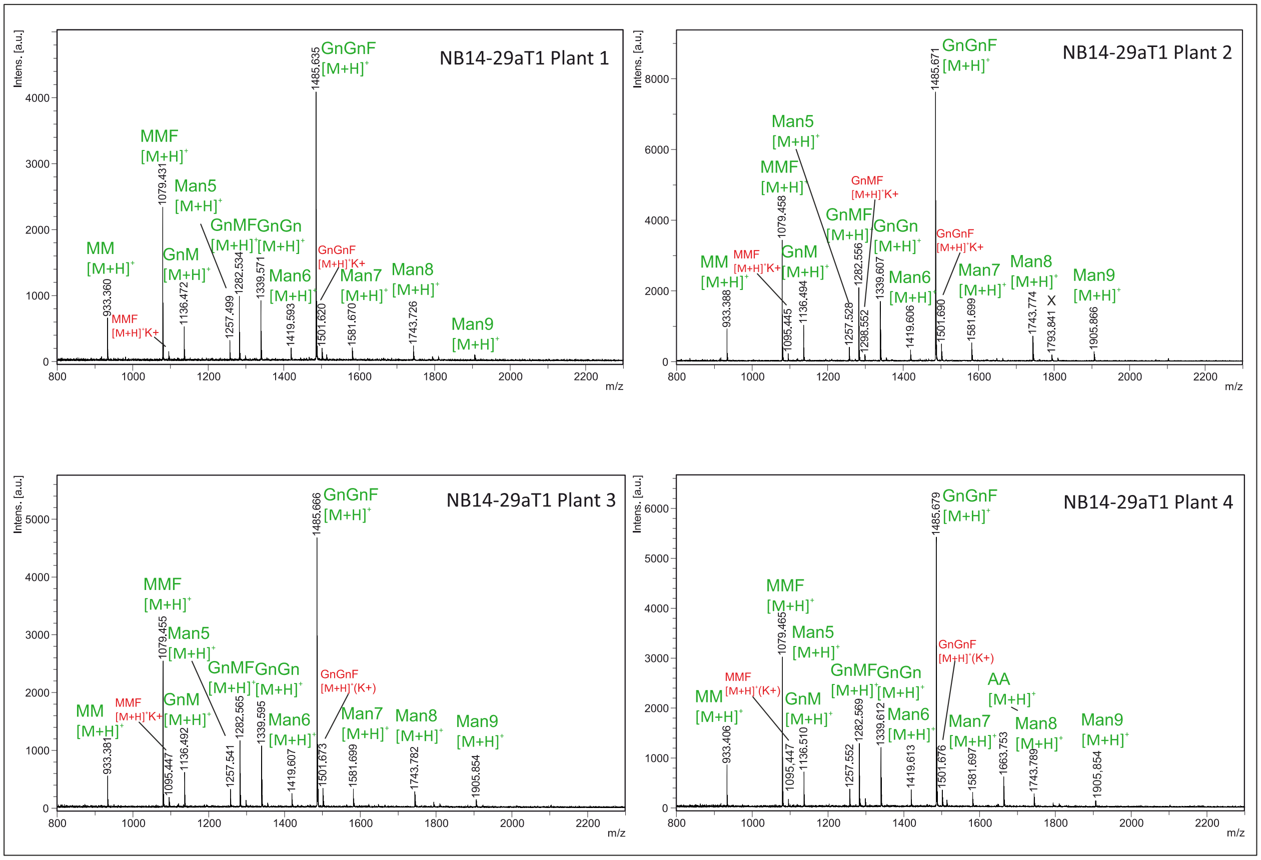
NB14-29aT1 Plant 1
NB14-29aT1 Plant 2
NB14-29aT1 Plant 3
NB14-29aT1 Plant 4
GnGnF
[M+H]+
MMF
Man5
GnMF
GnGn
GnM
MM
Man6
Man7
Man8
Man9
AA
Intens. [a.u.]
m/z

C

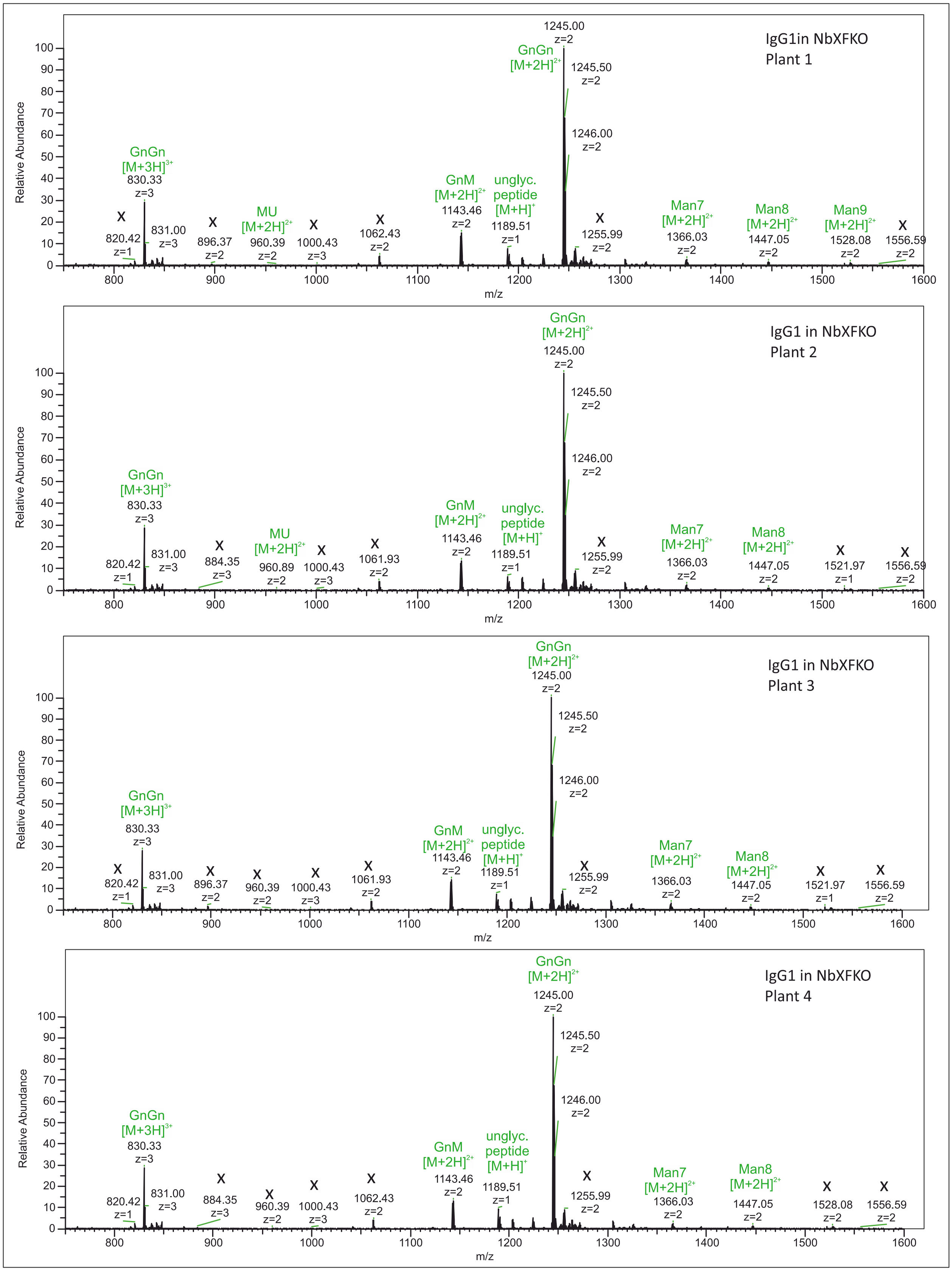

IgG1in NbXFKO
Plant 1
IgG1 in NbXFKO
Plant 2
IgG1 in NbXFKO
Plant 3
IgG1 in NbXFKO
Plant 4
GnGn
[M+2H]2+
1245.00
z=2
1245.50
z=2
1246.00
z=2
GnGn
[M+3H]3+
830.33
z=3
820.42
z=1
831.00
z=3
896.37
z=2
884.35
z=3
MU
[M+2H]2+
960.39
z=2
960.89
1000.43
z=3
1062.43
1061.93
GnM
[M+2H]2+
1143.46
z=2
unglyc.
peptide
[M+H]+
1189.51
z=1
1255.99
z=2
Man7
[M+2H]2+
1366.03
Man8
[M+2H]2+
1447.05
Man9
[M+2H]2+
1528.08
1521.97
1556.59
X
Relative Abundance
m/z

D

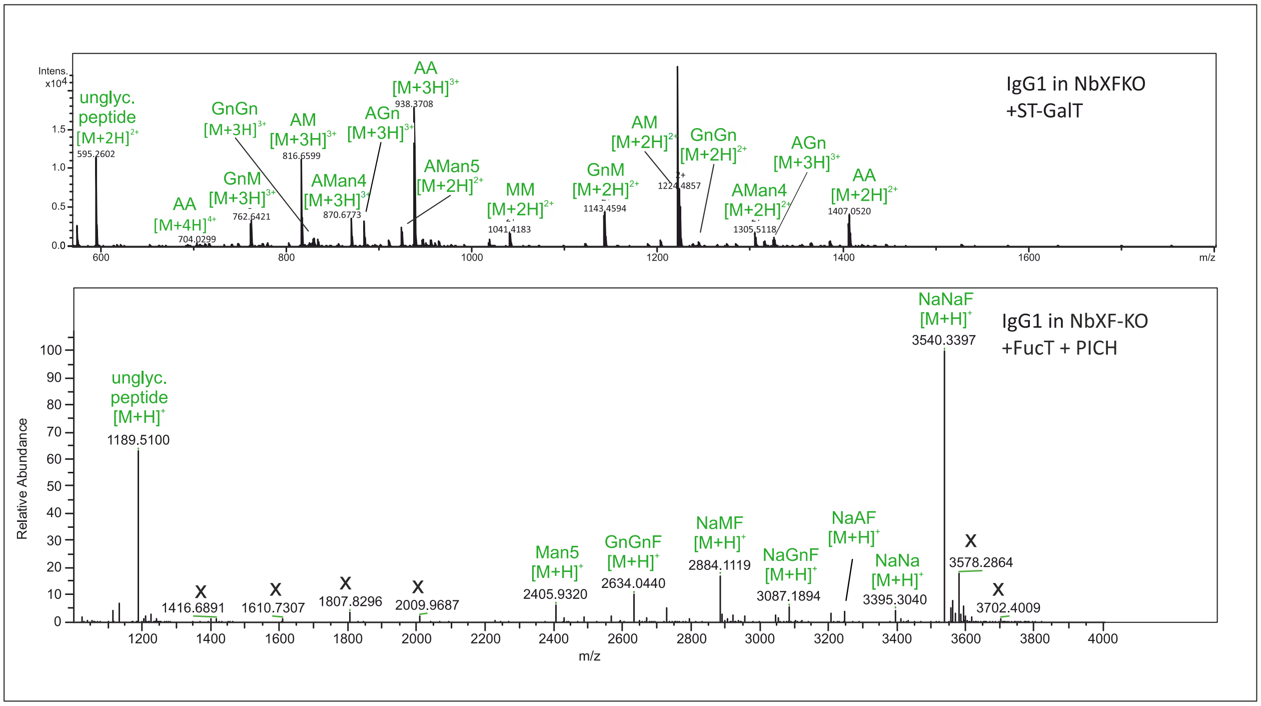

IgG1 in NbXFKO
+ST-GalT
unglyc.
peptide
[M+2H]2+
595.2602
AA
[M+4H]4+
704.0299
GnM
[M+3H]3+
762.6421
GnGn
[M+3H]3+
AM
[M+3H]3+
816.6599
AMan4
[M+3H]3+
870.6773
AGn
[M+3H]3+
AA
[M+3H]3+
938.3708
AMan5
[M+2H]2+
MM
[M+2H]2+
1041.4183
GnM
[M+2H]2+
1143.4594
AM
[M+2H]2+
1224.4857
GnGn
[M+2H]2+
AMan4
[M+2H]2+
1305.5118
AGn
[M+3H]3+
AA
[M+2H]2+
1407.0520
IgG1 in NbXF-KO
+FucT + PICH
Relative Abundance
m/z
unglyc.
peptide
[M+H]+
1189.5100
X
1416.6891
X
1610.7307
X
1807.8296
X
2009.9687
Man5
[M+H]+
2405.9320
GnGnF
[M+H]+
2634.0440
NaMF
[M+H]+
2884.1119
NaGnF
[M+H]+
3087.1894
NaAF
[M+H]+
NaNa
[M+H]+
3395.3040
NaNaF
[M+H]+
3540.3397
X
3578.2864
X
3702.4009

E

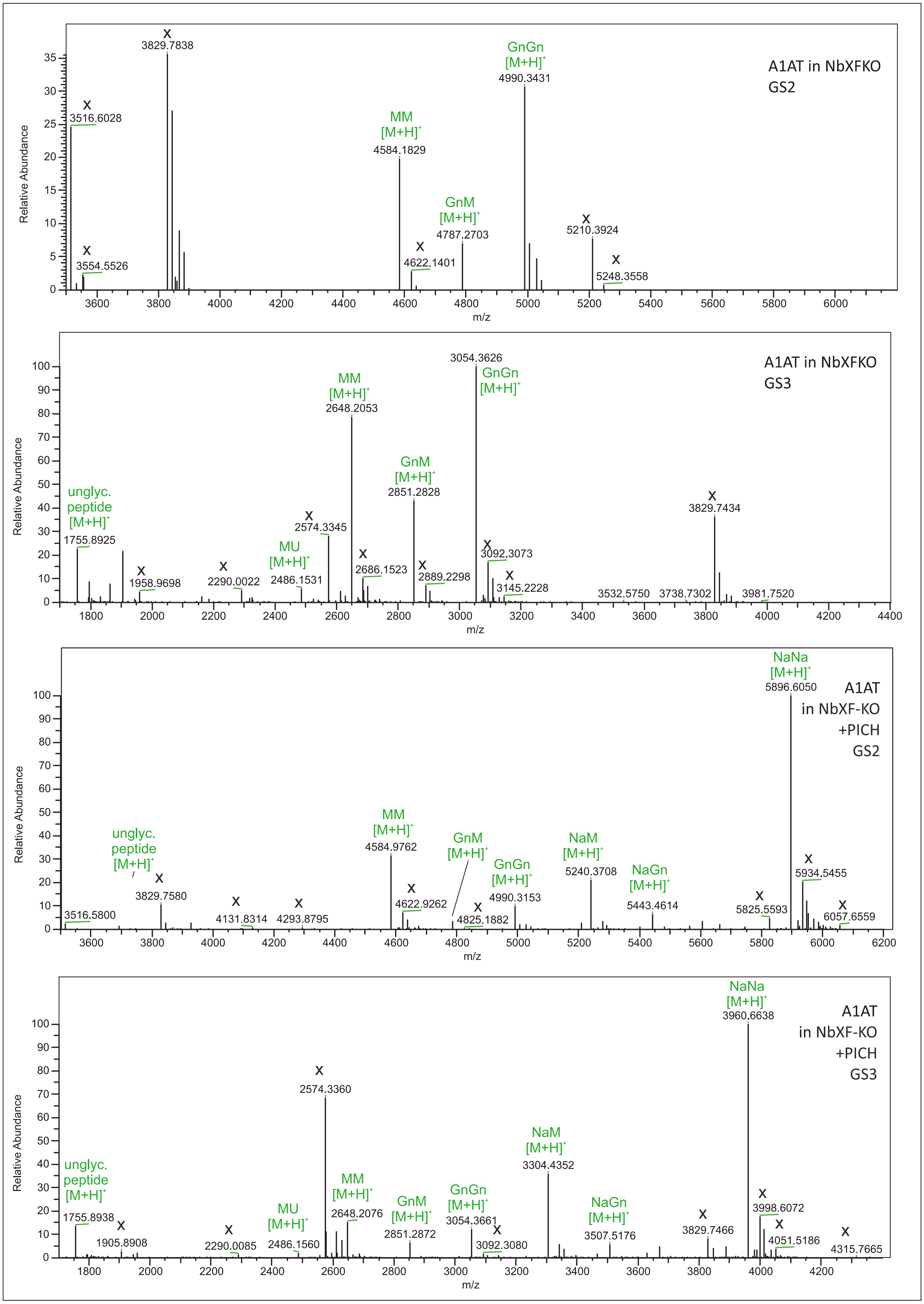

A1AT in NbXFKO
GS2
GnGn
[M+H]+
4990.3431
MM
[M+H]+
4584.1829
GnM
[M+H]+
4787.2703
x
3829.7838
x
3516.6028
x
3554.5526
x
4622.1401
x
5210.3924
x
5248.3558
Relative Abundance
m/z
A1AT in NbXFKO
GS3
3054.3626
GnGn
[M+H]+
MM
[M+H]+
2648.2053
GnM
[M+H]+
2851.2828
unglyc.
peptide
[M+H]+
1755.8925
MU
[M+H]+
2486.1531
x
2574.3345
x
1958.9698
x
2290.0022
x
2686.1523
x
2889.2298
x
3092.3073
x
3145.2228
3532.5750
3738.7302
x
3829.7434
3981.7520
A1AT
in NbXF-KO
+PICH
GS2
NaNa
[M+H]+
5896.6050
MM
[M+H]+
4584.9762
GnM
[M+H]+
GnGn
[M+H]+
4990.3153
NaM
[M+H]+
5240.3708
NaGn
[M+H]+
5443.4614
unglyc.
peptide
[M+H]+
3516.5800
x
3829.7580
x
4131.8314
x
4293.8795
x
4622.9262
x
4825.1882
x
5825.5593
x
5934.5455
x
6057.6559
A1AT
in NbXF-KO
+PICH
GS3
NaNa
[M+H]+
3960.6638
x
2574.3360
NaM
[M+H]+
3304.4352
unglyc.
peptide
[M+H]+
1755.8938
x
1905.8908
x
2290.0085
MU
[M+H]+
2486.1560
MM
[M+H]+
2648.2076
GnM
[M+H]+
2851.2872
GnGn
[M+H]+
3054.3661
x
3092.3080
NaGn
[M+H]+
3507.5176
x
3829.7466
x
3998.6072
x
4051.5186
x
4315.7665

F

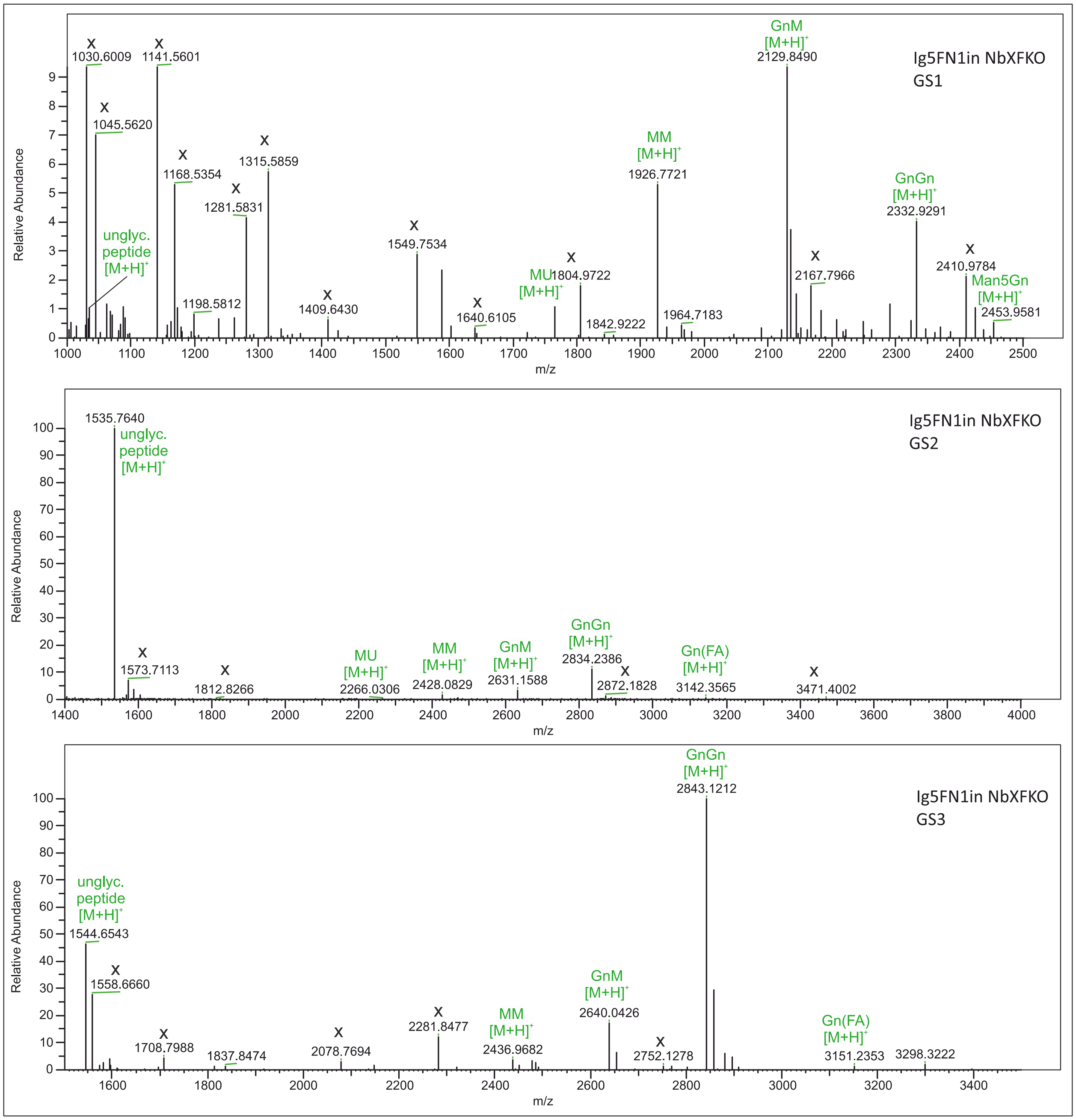
Ig5FN1in NbXFKO
GS1
Relative Abundance
m/z
unglyc.
peptide
[M+H]+
1030.6009
1141.5601
1045.5620
1168.5354
1281.5831
1315.5859
1198.5812
1409.6430
1549.7534
1640.6105
MU
[M+H]+
1804.9722
1842.9222
MM
[M+H]+
1926.7721
1964.7183
GnM
[M+H]+
2129.8490
2167.7966
GnGn
[M+H]+
2332.9291
2410.9784
Man5Gn
[M+H]+
2453.9581
Ig5FN1in NbXFKO
GS2
1535.7640
unglyc.
peptide
[M+H]+
1573.7113
1812.8266
MU
[M+H]+
2266.0306
MM
[M+H]+
2428.0829
GnM
[M+H]+
2631.1588
GnGn
[M+H]+
2834.2386
2872.1828
Gn(FA)
[M+H]+
3142.3565
3471.4002
Ig5FN1in NbXFKO
GS3
unglyc.
peptide
[M+H]+
1544.6543
1558.6660
1708.7988
1837.8474
2078.7694
2281.8477
MM
[M+H]+
2436.9682
GnM
[M+H]+
2640.0426
2752.1278
GnGn
[M+H]+
2843.1212
Gn(FA)
[M+H]+
3151.2353
3298.3222

G

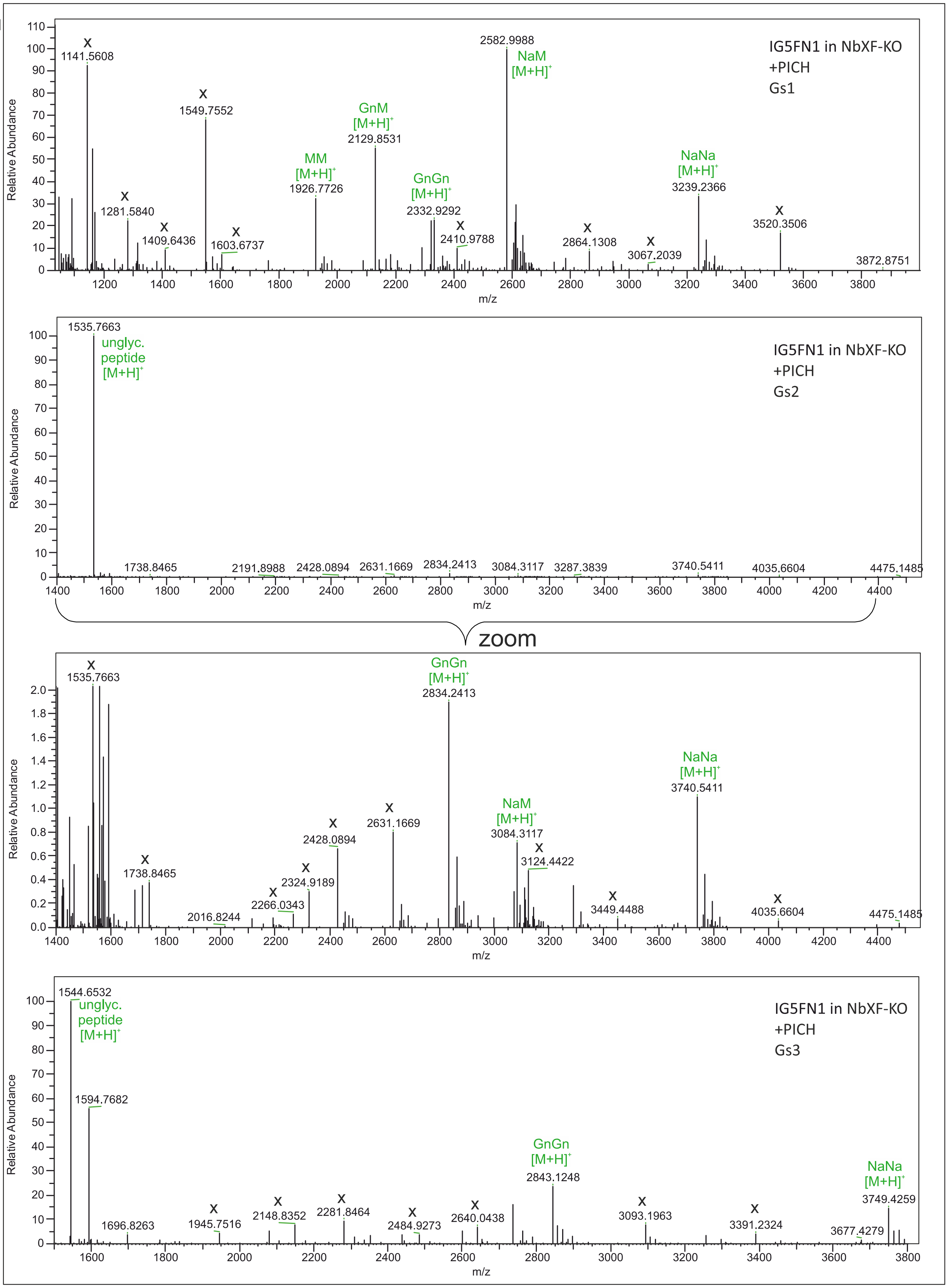
IG5FN1 in NbXF-KO
+PICH
Gs1
Relative Abundance
m/z
1141.5608
1281.5840
1409.6436
1549.7552
1603.6737
MM
[M+H]+
1926.7726
GnM
[M+H]+
2129.8531
GnGn
[M+H]+
2332.9292
2410.9788
2582.9988
NaM
[M+H]+
2864.1308
3067.2039
NaNa
[M+H]+
3239.2366
3520.3506
3872.8751
IG5FN1 in NbXF-KO
+PICH
Gs2
1535.7663
unglyc.
peptide
[M+H]+
1738.8465
2191.8988
2428.0894
2631.1669
2834.2413
3084.3117
3287.3839
3740.5411
4035.6604
4475.1485
zoom
1535.7663
1738.8465
2016.8244
2266.0343
2324.9189
2428.0894
2631.1669
GnGn
[M+H]+
2834.2413
NaM
[M+H]+
3084.3117
3124.4422
3449.4488
NaNa
[M+H]+
3740.5411
4035.6604
4475.1485
IG5FN1 in NbXF-KO
+PICH
Gs3
1544.6532
unglyc.
peptide
[M+H]+
1594.7682
1696.8263
1945.7516
2148.8352
2281.8464
2484.9273
2640.0438
GnGn
[M+H]+
2843.1248
3093.1963
3391.2324
3677.4279
NaNa
[M+H]+
3749.4259

H

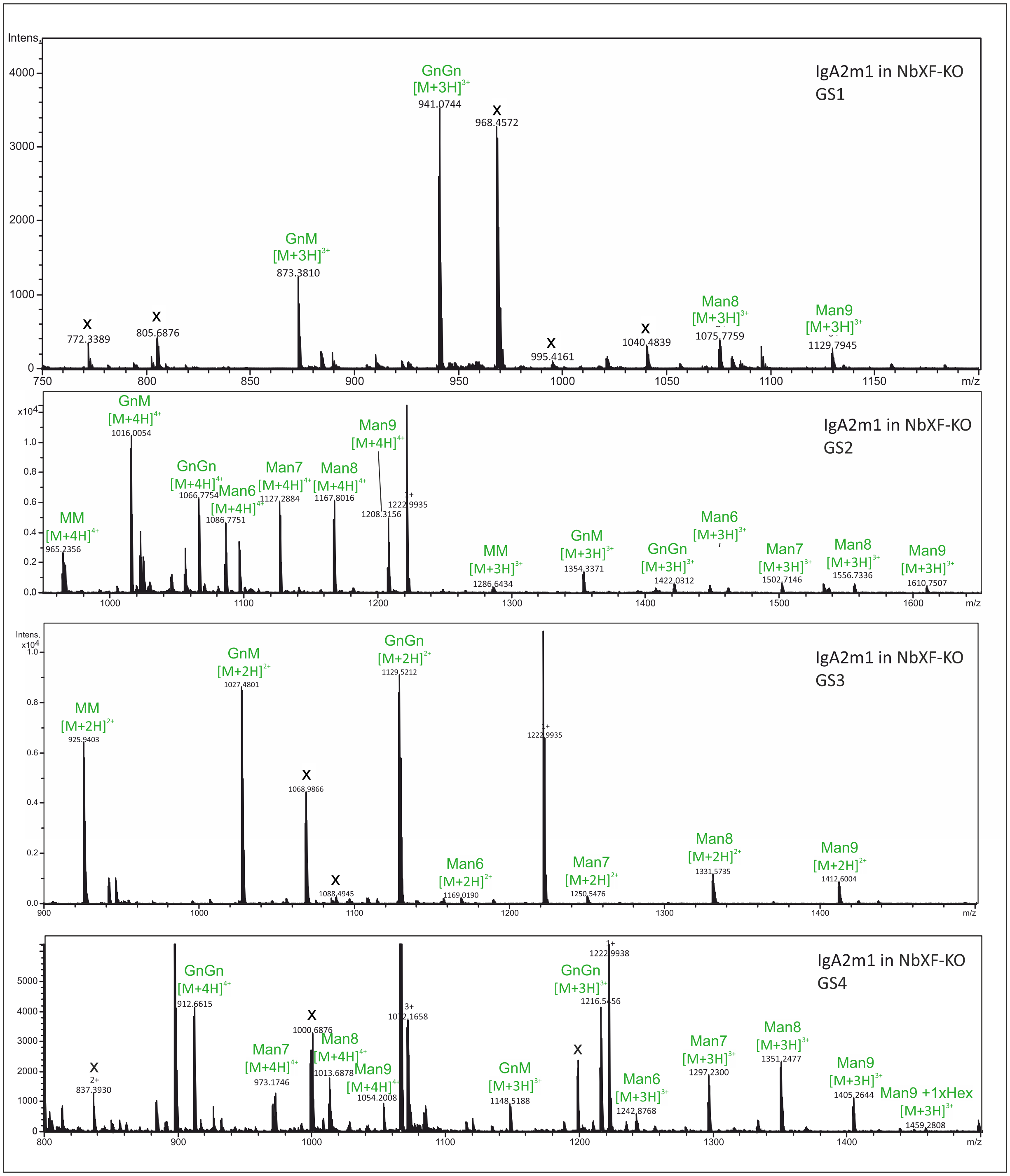

IgA2m1 in NbXF-KO
GS1
GnGn
[M+3H]3+
941.0744
968.4572
GnM
[M+3H]3+
873.3810
772.3389
805.6876
995.4161
1040.4839
Man8
[M+3H]3+
1075.7759
Man9
[M+3H]3+
1129.7945
Intens.
m/z
IgA2m1 in NbXF-KO
GS2
GnM
[M+4H]4+
1016.0054
MM
[M+4H]4+
965.2356
GnGn
[M+4H]4+
1066.7754
Man6
[M+4H]4+
1086.7751
Man7
[M+4H]4+
1127.2884
Man8
[M+4H]4+
1167.8016
Man9
[M+4H]4+
1208.3156
1+
1222.9935
MM
[M+3H]3+
1286.6434
GnM
[M+3H]3+
1354.3371
GnGn
[M+3H]3+
1422.0312
Man6
[M+3H]3+
Man7
[M+3H]3+
1502.7146
Man8
[M+3H]3+
1556.7336
Man9
[M+3H]3+
1610.7507
IgA2m1 in NbXF-KO
GS3
MM
[M+2H]2+
925.9403
GnM
[M+2H]2+
1027.4801
1068.9866
1088.4945
GnGn
[M+2H]2+
1129.5212
Man6
[M+2H]2+
1169.0190
1+
1222.9935
Man7
[M+2H]2+
1250.5476
Man8
[M+2H]2+
1331.5735
Man9
[M+2H]2+
1412.6004
IgA2m1 in NbXF-KO
GS4
2+
837.3930
GnGn
[M+4H]4+
912.6615
Man7
[M+4H]4+
973.1746
1000.6876
Man8
[M+4H]4+
1013.6878
Man9
[M+4H]4+
1054.2008
3+
1072.1658
GnM
[M+3H]3+
1148.5188
GnGn
[M+3H]3+
1216.5456
1+
1222.9938
Man6
[M+3H]3+
1242.8768
Man7
[M+3H]3+
1297.2300
Man8
[M+3H]3+
1351.2477
Man9
[M+3H]3+
1405.2644
Man9 +1xHex
[M+3H]3+
1459.2808

I

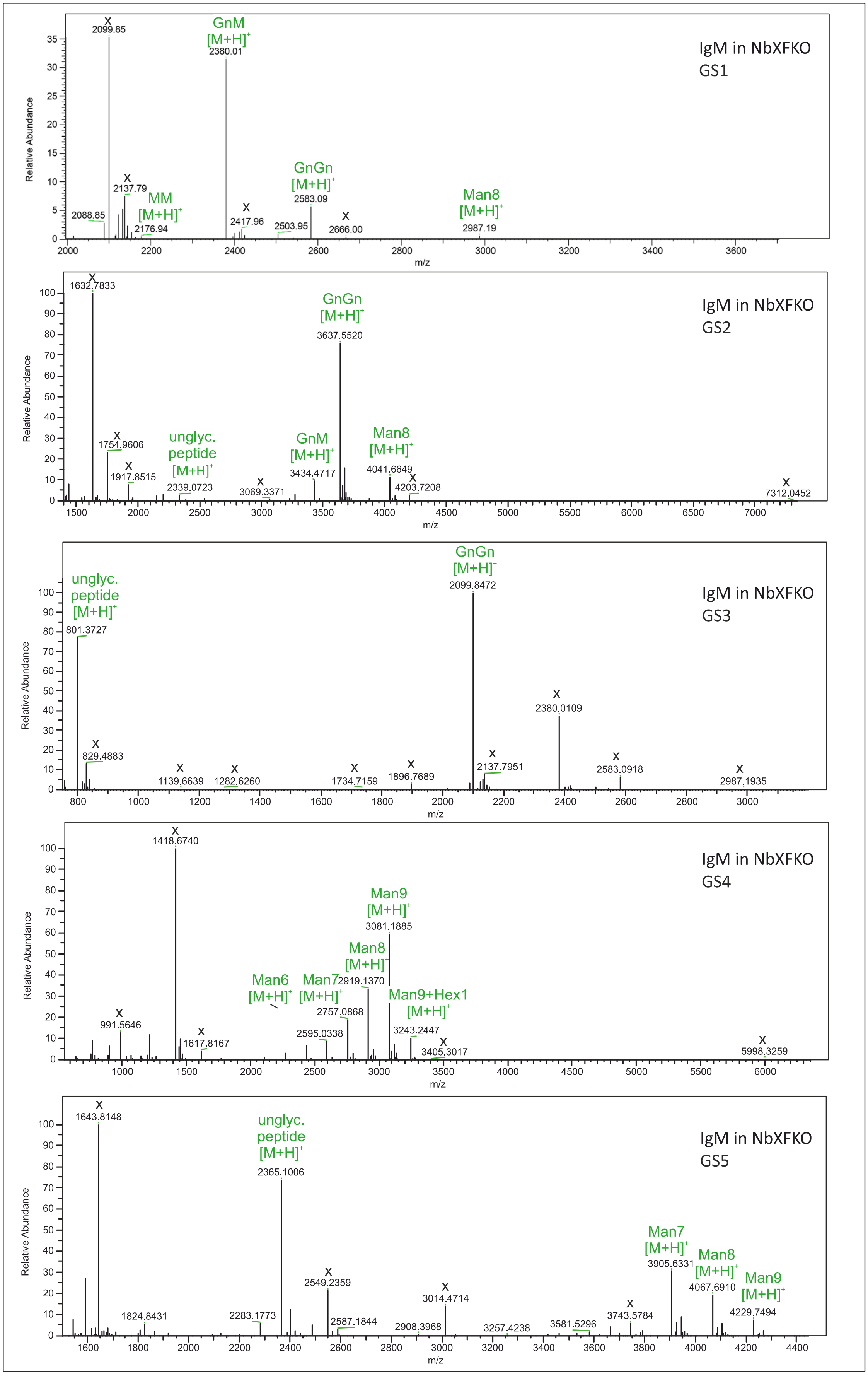
IgM in NbXFKO
GS1
GnM
[M+H]+
2380.01
2099.85
MM
[M+H]+
2176.94
GnGn
[M+H]+
2583.09
Man8
[M+H]+
2987.19
Relative Abundance
m/z
IgM in NbXFKO
GS2
1632.7833
GnGn
[M+H]+
3637.5520
unglyc.
peptide
[M+H]+
2339.0723
GnM
[M+H]+
3434.4717
Man8
[M+H]+
4041.6649
7312.0452
IgM in NbXFKO
GS3
unglyc.
peptide
[M+H]+
801.3727
GnGn
[M+H]+
2099.8472
2380.0109
IgM in NbXFKO
GS4
1418.6740
Man6
[M+H]+
Man7
[M+H]+
2757.0868
Man8
[M+H]+
2919.1370
Man9
[M+H]+
3081.1885
Man9+Hex1
[M+H]+
3243.2447
IgM in NbXFKO
GS5
1643.8148
unglyc.
peptide
[M+H]+
2365.1006
Man7
[M+H]+
3905.6331
Man8
[M+H]+
4067.6910
Man9
[M+H]+
4229.7494

J

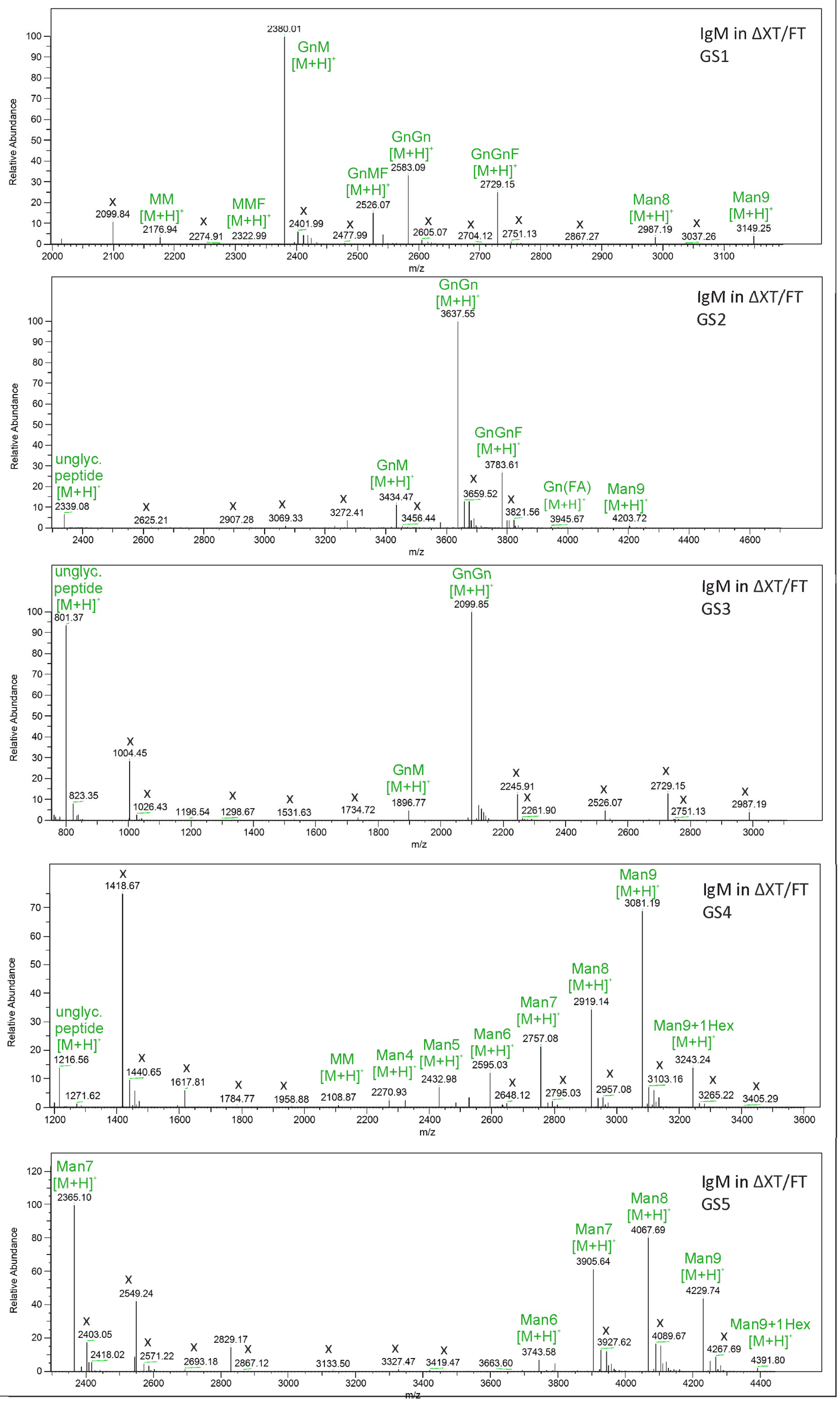
IgM in ΔXT/FT
GS1
GnM [M+H]+ 2380.01
MM [M+H]+ 2176.94
MMF [M+H]+ 2322.99
GnMF [M+H]+ 2526.07
GnGn [M+H]+ 2583.09
GnGnF [M+H]+ 2729.15
Man8 [M+H]+ 2987.19
Man9 [M+H]+ 3149.25
x 2099.84
x 2274.91
x 2401.99
x 2477.99
x 2605.07
x 2704.12
x 2751.13
x 2867.27
x 3037.26
IgM in ΔXT/FT
GS2
unglyc. peptide [M+H]+ 2339.08
GnM [M+H]+ 3434.47
GnGn [M+H]+ 3637.55
GnGnF [M+H]+ 3783.61
Gn(FA) [M+H]+ 3945.67
Man9 [M+H]+ 4203.72
x 2625.21
x 2907.28
x 3069.33
x 3272.41
x 3456.44
x 3659.52
x 3821.56
IgM in ΔXT/FT
GS3
unglyc. peptide [M+H]+ 801.37
GnM [M+H]+ 1896.77
GnGn [M+H]+ 2099.85
823.35
x 1004.45
x 1026.43
1196.54
x 1298.67
x 1531.63
x 1734.72
x 2245.91
x 2261.90
x 2526.07
x 2729.15
x 2751.13
x 2987.19
IgM in ΔXT/FT
GS4
unglyc. peptide [M+H]+ 1216.56
MM [M+H]+ 2108.87
Man4 [M+H]+ 2270.93
Man5 [M+H]+ 2432.98
Man6 [M+H]+ 2595.03
Man7 [M+H]+ 2757.08
Man8 [M+H]+ 2919.14
Man9 [M+H]+ 3081.19
Man9+1Hex [M+H]+ 3243.24
1271.62
x 1418.67
x 1440.65
x 1617.81
x 1784.77
x 1958.88
x 2648.12
x 2795.03
x 2957.08
x 3103.16
x 3265.22
x 3405.29
IgM in ΔXT/FT
GS5
Man7 [M+H]+ 2365.10
Man6 [M+H]+ 3743.58
Man7 [M+H]+ 3905.64
Man8 [M+H]+ 4067.69
Man9 [M+H]+ 4229.74
Man9+1Hex [M+H]+ 4391.80
x 2403.05
2418.02
x 2549.24
x 2571.22
x 2693.18
2829.17
x 2867.12
x 3133.50
x 3327.47
x 3419.47
3663.60
x 3927.62
x 4089.67
x 4267.69
Relative Abundance
m/z

K

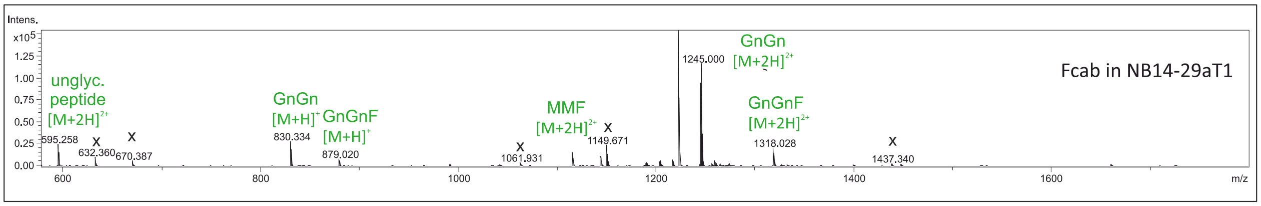

Figure S8 MS spectra used for relative quantification of N-glycans. **(A)** Total glycans extracted from NbXF-KO and **(B)** NB14-29aT1 measured by MALDI-TOF. Ions are Na+ ionized if not indicated otherwise (K+ ionized are depicted in red and were not considered for quantification as they appear in same the same ratio); **(C)** LC-ESI-MS profiles/spectra from IgG1-Fc (Rituximab) glycopeptide (derived from plant 1-4, **(D)** Rituximab co-infiltrated with ST-GalT (top) and FucT + pICH 88266 [17] (bottom, deconvoluted profile), respectively; (**E-J**) LC-ESI-MS profiles/spectra from various reporter proteins (all deconvoluted profiles except IgA2m1); (**K**) Fcab expressed in NB14-29aT1. Peptide sequence and molecular sites are depicted in Table S8. A1AT, Ig5FN1 and IgM are shown deconvoluted (see MM). Glycoforms are depicted in green including their charge state. x indicates non glycopeptide peaks.

	**Batch 1**	**Batch 2**	**Batch 3**
**N-Glycan Profile**<br>Complex<br>Mannosidic<br>Others	2%<br>11%<br>87%	1%<br>15%<br>83%	2%<br>10%<br>88%
**Leaf material (g)**	24	38	40
**Yield of purified IgG1 (mg/kg)**	300	220	250

Figure S9 IgG1 expression profile of three independent NbXF-KO infiltration experiments.

References

1. Uetz P, Melnik S, Grunwald-Gruber C, Strasser R, Stoger E. CRISPR/Cas9-mediated knockout of a prolyl-4-hydroxylase subfamily in Nicotiana benthamiana using DsRed2 for plant selection. Biotechnology Journal. 2022;17(7).
2. Cermak T, Curtin SJ, Gil-Humanes J, Cegan R, Kono TJY, Konecna E, et al. A Multipurpose Toolkit to Enable Advanced Genome Engineering in Plants. Plant Cell. 2017;29(6):1196-217.
3. Kapelski S, Boes A, Spiegel H, de Almeida M, Klockenbring T, Reimann A, et al. Fast track antibody V-gene rescue, recombinant expression in plants and characterization of a PfMSP4-specific antibody. Malar J. 2015;14:50.
4. Li J, Stoddard TJ, Demorest ZL, Lavoie PO, Luo S, Clasen BM, et al. Multiplexed, targeted gene editing in Nicotiana benthamiana for glyco-engineering and monoclonal antibody production. Plant Biotechnol J. 2016;14(2):533-42.
5. Strasser R, Stadlmann J, Schahs M, Stiegler G, Quendler H, Mach L, et al. Generation of glyco-engineered Nicotiana benthamiana for the production of monoclonal antibodies with a homogeneous human-like N-glycan structure. Plant Biotechnol J. 2008;6(4):392-402.
6. Yau Y-Y, Easterling M, Brennan L. Rapid Agrobacterium mediated transformation of tobacco cotyledons using toothpicks. bioRxiv. 2017:204891.
7. Goritzer K, Maresch D, Altmann F, Obinger C, Strasser R. Exploring Site-Specific N-Glycosylation of HEK293 and Plant-Produced Human IgA Isotypes. J Proteome Res. 2017;16(7):2560-70.
8. Kallolimath S, Palt R, Föderl-Höbenreich E, Sun L, Chen Q, Pruckner F, et al. Glyco-engineered pentameric SARS-CoV-2 IgMs show superior activities compared to IgG1 orthologues. Frontiers in Immunology. 2023.
9. Jez J, Antes B, Castilho A, Kainer M, Wiederkum S, Grass J, et al. Significant impact of single N-glycan residues on the biological activity of Fc-based antibody-like fragments. J Biol Chem. 2012;287(29):24313-9.
10. Castilho A, Windwarder M, Gattinger P, Mach L, Strasser R, Altmann F, et al. Proteolytic and N-glycan processing of human alpha1-antitrypsin expressed in Nicotiana benthamiana. Plant Physiol. 2014;166(4):1839-51.
11. Kallolimath S, Castilho A, Strasser R, Grunwald-Gruber C, Altmann F, Strubl S, et al. Engineering of complex protein sialylation in plants. Proc Natl Acad Sci U S A. 2016;113(34):9498-503.
12. Kallolimath S, Gruber C, Steinkellner H, Castilho A. Promoter Choice Impacts the Efficiency of Plant Glyco-Engineering. Biotechnol J. 2018;13(1).
13. Castilho A, Gruber C, Thader A, Oostenbrink C, Pechlaner M, Steinkellner H, et al. Processing of complex N-glycans in IgG Fc-region is affected by core fucosylation. Mabs. 2015;7(5):863-70.
14. Strasser R, Altmann F, Mach L, Glossl J, Steinkellner H. Generation of Arabidopsis thaliana plants with complex N-glycans lacking beta1,2-linked xylose and core alpha1,3-linked fucose. FEBS Lett. 2004;561(1-3):132-6.
15. Blasco H, Lalmanach G, Godat E, Maurel MC, Canepa S, Belghazi M, et al. Evaluation of a peptide ELISA for the detection of rituximab in serum. J Immunol Methods. 2007;325(1-2):127-39.
16. Chen B, Gilbert LA, Cimini BA, Schnitzbauer J, Zhang W, Li GW, et al. Dynamic imaging of genomic loci in living human cells by an optimized CRISPR/Cas system. Cell. 2013;155(7):1479-91.
17. Izadi S, Kunnummel V, Steinkellner H, Werner S, Castilho A. Assessment of transient expression strategies to sialylate recombinant proteins in N. benthamiana. J Biotechnol. 2023;365:48-53.
